# ﻿New species and a fascinating diversity of Chironomidae (Diptera, Insecta) in and around an overlooked urban vernal pool

**DOI:** 10.3897/zookeys.1208.124495

**Published:** 2024-07-29

**Authors:** Armin Namayandeh, Sergio Guerra, Natasha Islam, Taylor James, Patrick L. Hudson, Edris Ghaderi, Thameena Yusuf, Adrian A. Vasquez, Jeffrey L. Ram

**Affiliations:** 1 Department of Environmental and Life Sciences, Trent University, 1600 West Bank Drive, Peterborough, ON, Canada; 2 Department of Physiology, School of Medicine, Wayne State University, Detroit, MI 48201, USA; 3 5597 New Meadow Drive, Ypsilanti, MI 48197, USA; 4 Department of Fisheries Sciences, Faculty of Natural Resources, University of Kurdistan, Sanandaj, Iran; 5 Department of Fisheries and Aquatic Ecology, Faculty of Fisheries and Environmental Sciences, Gorgan University of Agricultural Sciences and Natural Resources, Gorgan, Iran; 6 Department of Biology, College of Liberal Arts and Sciences, Mercer University, 1501 Mercer University Drive, Macon, GA 31207, USA; 7 Department of Physiology, School of Medicine, Wayne State University, Detroit, MI 48201, USA

**Keywords:** *Bryophaenocladius*, Detroit, educational outreach, faunistic records, *Limnophyes*, *Rheocricotopus*, temporary aquatic habitats, urban park

## Abstract

In this study, the biodiversity of Chironomidae was investigated in Palmer Park Pond A, an urban vernal pond in Detroit, Michigan, USA. This study is developed as part of our ongoing Public Environmental Outreach Program at the Detroit Exploration and Nature Center in Palmer Park. Twenty-one Chironomidae species were discovered in and on the adjacent riparian vegetation of this pond using molecular and morphological methods. Three species *Bryophaenocladiuspalmerparcum* Namayandeh & Hudson **sp. nov.**, *Limnophyesstagnum* Namayandeh, Guerra & Ram **sp. nov.**, and *Rheocricotopus* (*s. s.*) *angustus* Namayandeh & Hudson **sp. nov.** are new to science. *Bryophaenocladiuspalmerparcum***sp. nov.** and *L.stagnum***sp. nov.** are unusual Orthoclads, with *B.palmerparcum***sp. nov.** possessing a setose, short, and wide anal point and *L.stagnum***sp. nov.** lacking lanceolate setae on both sexes. Based on the shape of superior volsella, *R.angustus***sp. nov.**, belongs to the *effusus* group, which was also confirmed by DNA barcoding molecular analysis. In this study, a new faunistic record was also found for the Nearctic as well as four new faunistic records for the state of Michigan. Ephemeral aquatic habitats such as vernal pools are often overlooked or destroyed by urbanization activities, controlling vector species, creating groomed fields, and/or residential development. Therefore, finding these new species demonstrates the biodiversity value of vernal ponds as important habitats, further motivating us to preserve them.

## ﻿Introduction

Vernal pools are small, shallow, isolated bodies of water occurring ephemerally in or in relation to the woodlands that surround them. Their hydrologic regime is driven by the seasonality of precipitation (Colburn 2004), the surrounding land surface’s relief and drainage characteristics, and the surrounding vegetation’s evapotranspiration (Cartwright et al. 2022). Despite their ephemeral and isolated nature, they display a remarkable and distinct biodiversity of fauna, especially invertebrates. Among invertebrates, aquatic insects, particularly Chironomidae species, are an ever-present feature of these habitats. A long-term study of Sunfish Pond, a vernal pond in southern Ontario, produced some 98 invertebrate taxa, with Chironomidae present in almost all hydrologic phases of the pond (Williams 2006). A species of *Einfeldia* occurred during the entire aquatic phase of the ponds. Other species occurred during the first few days of spring, such as those in *Trissocladius*, *Eukiefferiella*, *Phaenopsectra*, *Parachironomus*, and *Polypedilum*. Some species appeared within the first few weeks of the pond formation, such as the species of *Micropsectra*, *Corynoneura*, *Ablabesmyia*, and *Psectrotanypus*, and others appeared before ponds dried up, such as the species of *Cricotopus*. Williams (2006) reported no Chironomidae during the drought cycle of Sunfish Pond. However, we know from other studies that many species of Chironomidae can resist the drought in temporary habitats either by cryptobiosis, diapause, cocoon forming, or by migrating to the deeper part of the substrate or hyporheic zone (Hinton 1960; Jones 1975; Grodhaus 1976; Delettre 1986; McLachlan 1988; Frouz et al. 2003; Suemoto et al. 2004; Cornette et al. 2022).

Temporary habitats such as vernal pools are often overlooked, as the value of biodiversity conservation is usually prioritized for exotic places (DeGasparro et al. 2020). Many times, new faunistic records and biogeographical gaps in species distribution can be filled by studying overlooked habitats, especially in places regarded as disturbed, such as farms and natural habitats of urban areas (Owen and Owen 1975; Quistberg et al. 2016; Namayandeh and Beresford 2017; DeGasparro et al. 2020; Griffiths-Lee et al. 2022). An advantage of urban natural habitats is that scientists can engage and motivate the public in the collection of specimens occurring closer to home (Dearborn and Kark 2010). Furthermore, researchers can provide environmental education to people with no background or specialization, such as high school and undergraduate students. The ecological importance of temporary habitats can be seen by emphasis on their value as refugia or connecting habitat patches with metacommunities and metapopulations. In this mosaic of connecting habitats, the disturbance rate (e.g., drought) can determine the expansion or reduction of species populations. However, local extinction does not necessarily cause the regional population to be exterminated (Larned et al. 2010).

We developed this study as part of our ongoing Public Environmental Outreach Program at Detroit Exploration and Nature Center (DEN), located at the northern edge of Palmer Park, Detroit, Michigan. We also engaged and trained four premed students from Wayne State University to perform DNA extractions, amplification, sequence analysis, and PCR as part of our educational outreach goals. The study area, Palmer Park, is a 200-hectare managed urban park that contains a primary (i.e., virgin) forest. The area surrounding Palmer Park is a typical built-up urban environment. Within the woods, there are many naturally occurring vernal ponds. One, in particular, is a sizeable vernal pool that we have monitored for nearly two years, during 2022–2023, named Palmer Park Pond A, referred to hereafter as Pond A.

Despite its ephemeral nature, we collected 20 Chironomidae species in and around (i.e., riparian zone) this overlooked urban habitat. Three species, *Bryophaenocladiuspalmerparcum* sp. nov., *Limnophyesstagnum* sp. nov., and *Rheocricotopus* (*s. s.*) *angustus* sp. nov. are new to science. Additionally, we report one new faunistic record for the Nearctic and four new faunistic records for Michigan. We also found and described the adult male of a morphospecies, likely to be the Chironomussp.parariparius described by Martin (2023). These records further improve the existing biogeographical gaps for the distribution of Chironomidae species in the Nearctic and the Holarctic.

## ﻿Materials and methods

### ﻿Study area

**Figure 1.**
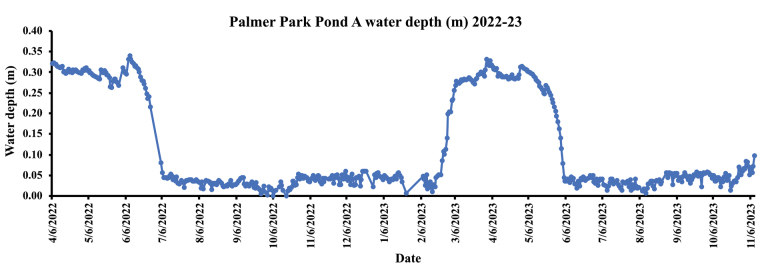
A hydrograph of Palmer Park Pond A water depth (m) 2022–23.

**Figure 2.**
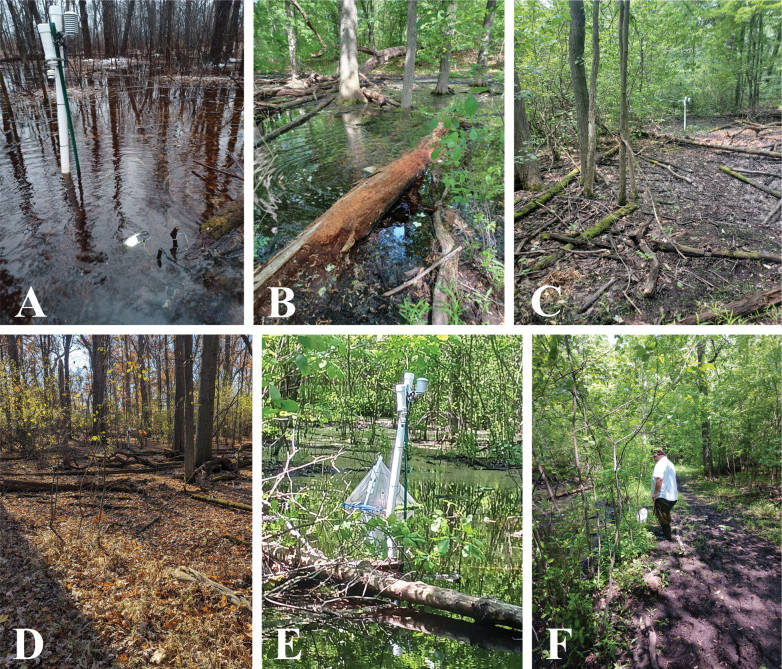
The study habitat, Palmer Park Pond A, Detroit, Michigan, USA **A** wet phase in late March **B** wet phase in late May **C** dry phase in late June **D** dry phase in November **E** emergence trap **F** collecting adults using sweep nets on riparian vegetation.

Vernal Pond A is located in Witherell Woods, 90 acres of virgin forest in Palmer Park, elevation ca 190 m, 42.42766°N, 83.11741°W. Based on the visual observation of the water level displayed in a hydrograph obtained from the pond, 2022–23, the pond’s wet phase (i.e., period) of the pond starts in mid to late winter, March or early April, and could last until late June. However, depending on winter and spring precipitation, the pond could dry up as early as late May or early June. The dry phase starts mid-summer and lasts until early winter, January, or February (Figs 1, 2A–D).

### ﻿Sampling collection, preparation imagery, and faunistic records

We collected the aquatic larvae and the emerging adults from and around Pond A. Larvae were collected using a hand-held net and kicking the substrate for 3 minutes in random habitats in the pond. The net contents were emptied and thoroughly washed into a 6 mm mesh-size sieve mounted on a 250-µm mesh sieve. We emptied the content of the 250-µm sieve into a 473-ml bottle and placed samples in an ice chest for transport to the lab for sorting within one to two days. For longer-term preservation, 90% ethanol was added to the collected organisms. Adults were collected with a self-built emergence similar to the design of Cadmus et al. (2016) and by sweep net from the vegetation around the pond’s edges preserved in 90% ethanol (Fig. 2E, F). We sorted the adults and immatures using a sorting scope and mounted them using a procedure outlined in Namayandeh and Hudson (2022). The depth of the pond was measured using a Dragino LDDS20 LoRaWAN Liquid Level Sensor, installed in the deepest part of the pond. The depth was measured from the water surface to the sensor, which was very close to the pond’s substrate. We corrected the values that were considered noise in the data, defined as data obtained due to the instrument’s temporary malfunction, for instance, temporary blockage by leaf litter, sediment, or invertebrates.

The imagery was produced using a Diagnostic Instruments Inc. Spot 5.1 camera mounted on an Olympus BX51 compound scope. The illustrations were produced based on the obtained images using Inkscape 1.2.2(2022): Draw Freely software. Morphological terminology, abbreviation, and measurements follow those of Sæther (1977, 1980).

The locations and depositories of species are as follows: Michigan State University, the Albert J. Cook Arthropod Research Collection (**ARC**); Canadian National Collection of Insects, Arachnids and Nematodes, Ottawa, Canada (**CNC**); Centre for Biodiversity Genomics, University of Guelph, Canada (**CBG**); private collection of Patrick L. Hudson, Ypsilanti, Michigan, USA (**PLH**); private collection of Thomas Bendt, Heyerhütte, Germany (**TB**).

We determined the new records by examining all available catalogs, such as those of Ashe and O’Connor (2012), Bright (2024), Caldwell et al. (1997), Cranston and Oliver (1988), and Oliver et al. (1990). Additionally, based on the DNA sequences we obtained in this study, we examined all matching sequences in BOLD and GenBank and their corresponding geographical location.

### ﻿Molecular and phylogenetic analyses

We could only extract sufficient DNA for amplification and successful sequencing from ten of 20 species in this study. The condition of the tissues and scarcity of specimens prevented us from either extracting DNA or dedicating the whole animal tissue to the molecular barcoding procedure described below. Genomic DNA was extracted from the full tissues of the adults and larval Chironomidae using the Qiagen DNA Blood and Tissue Kit (Qiagen, Inc., Germantown, MD) as described previously by Failla et al. (2016) and Vasquez et al. (2022). A 658 base pair fragment of the cytochrome oxidase subunit 1 (COI) was amplified using the universal primers LCO1490 and HCO2198 (Folmer et al. 1994). DNA amplification was carried out in 20 μl reactions using GoTaq DNA polymerase (Promega Co., Madison, WI), 1× manufacturer’s buffer, 10 mM dNTP mix, 10 mM of each primer, and 200–250 ng template DNA. The amplification cycles were performed using an initial denaturation step of 95 °C for five min, followed by 34 cycles of 94 °C for 30 s, 51 °C for 30 s, 72 °C for one min, and a final extension at 72 °C for three min. The amplicons were shipped to GENEWIZ, a subsidiary of Azenta Life Sciences, for Sanger Sequencing. Sequence traces were evaluated and edited with Chromas 2024 (http://www.technelysium.com.au/chromas.html). We submitted new sequences to the BOLD database (http://dx.doi.org/10.5883/DS-DTPPA). The list of sequences, codes, GenBank, or BOLD accessions is provided in Suppl. material 1: table S1.

Phylogenetic trees based on COI sequences were created using Neighbour-Joining (NJ) and Maximum Likelihood (ML) methods. The NJ phylogenetic tree was made using Kimura’s 2-parameter (K2P) model in MEGA X with 10000 bootstrap replications (Kumar et al. 2018). To construct the tree using ML, sequences were aligned using Clustal X v. 2.1 software (Larkin et al. 2007). The resulting alignment was analyzed in jModelTest software v. 2.1.7 (Darriba et al. 2012) to determine the optimal model of molecular evolution and gamma rate heterogeneity using the AIC. We constructed the ML trees using RAxMLHPC BlackBox (8.2.12) software (Stamatakis 2014) in the CIPRES Scientific Gateway v. 3.3 XSED (Miller et al. 2012) and with 10000 Bootstrap repeats. Trees constructed in the ML model were visualized in FigTree v. 1.4.2 (Rambaut 2014). *Culicoidessanguisuga* Gornostaeva, 1977, *Bryophaenocladiusscanicus* (Brundin, 1947), and *Chironomus* (*s. s.*) *maturus* Johannsen, 1908 sequences obtained from BOLD were used as out group in phylogenetic trees. We obtained the phylogenetic distances using Kimura-2-parameter (K2P) model (Kimura 1980) in MEGA X (Kumar et al. 2018). We first determined the intraspecific K2P distance between the sequences from this study and the intraspecific K2P distance between sequences available in BOLD and NCBI.

To determine the limits of “molecular species” we used Automatic Barcode Gap Discovery (ABGD) (Puillandre et al. 2012). (https://bioinfo.mnhn.fr/abi/public/abgd/abgdweb.html) and K2P distances, which we considered 5% as a threshold value. We ran ABGD with P min = 0.001, P max = 0.1, and a gap width of 1.5, all for a total of 10 steps setting to calculate the barcode gap in the distribution of pairwise differences.

## ﻿Results

### ﻿Molecular and phylogenetic analyses

The analyses of NJ and ML on sequences of Chironomidae from Pond A and those obtained from NCBI and BOLD produced different tree topologies, with NJ more appropriately separating the genera into appropriate subfamilies (Suppl. material 1: fig. S1). Based on the NJ analysis, *Chironomus* (*s. s.*) *acidophilus* Keyl, 1960, represented by a single sequence barcode clustered with two voucher sequences of *C.acidophilus*, accession numbers KR663763 and HQ581839, from Ontario, Canada (Hebert et al. 2016). The two sequences of *Chironomus* (*s. s.*) *maturus* Johannsen, 1908 from Pond A clustered with voucher sequences of *C.maturus*, accession numbers MF707093 and HQ581849, from Ontario, Canada (de Waard et al 2019). The single sequence of *Tanytarsus* we identified as *Tanytarsusguerlus* (Roback, 1957) clustered with two sequences of *T.guerlus*, accession numbers KR657911, and KR638783 from Ontario, Canada (Hebert et al. 2016). The two sequences of *Polypedilum* we identified as *Polypedilum* (*s. s.*) sp. clustered with sequences identified only as *Polypedilum* sp., accession numbers HQ982463 and HQ981830, from Ontario, Canada. The single sequence of *Smittiaterrestris* from Pond A clustered with sequence of *Smittia* species accession number HQ582868, and *Smittiaterrestris* (= *Bryophaenocladiusterrestris*), accession number OP927437 from Rathenow-Grütz, Germany (Chimeno et al. 2023), and accession numbers HQ582868, HQ981435, from Arkansas, USA, and Ontario, Canada.

The average intraspecific K2P distance between the specimens of *C.maturus* was 0.002 (0.20%), for the specimens of *C.acidophilus* 0.001 (0.1%), for *T.guerlus* 0.001 (0.1%) and for *S.terrestris* 0.002 (0.2%). These results further confirm the new faunistic records for Michigan and the Nearctic. Kimura 2-Parameter (K2P) average interspecific distances for all Pond A species is provided in Suppl. material 1: table S3.

**Figure 3.**
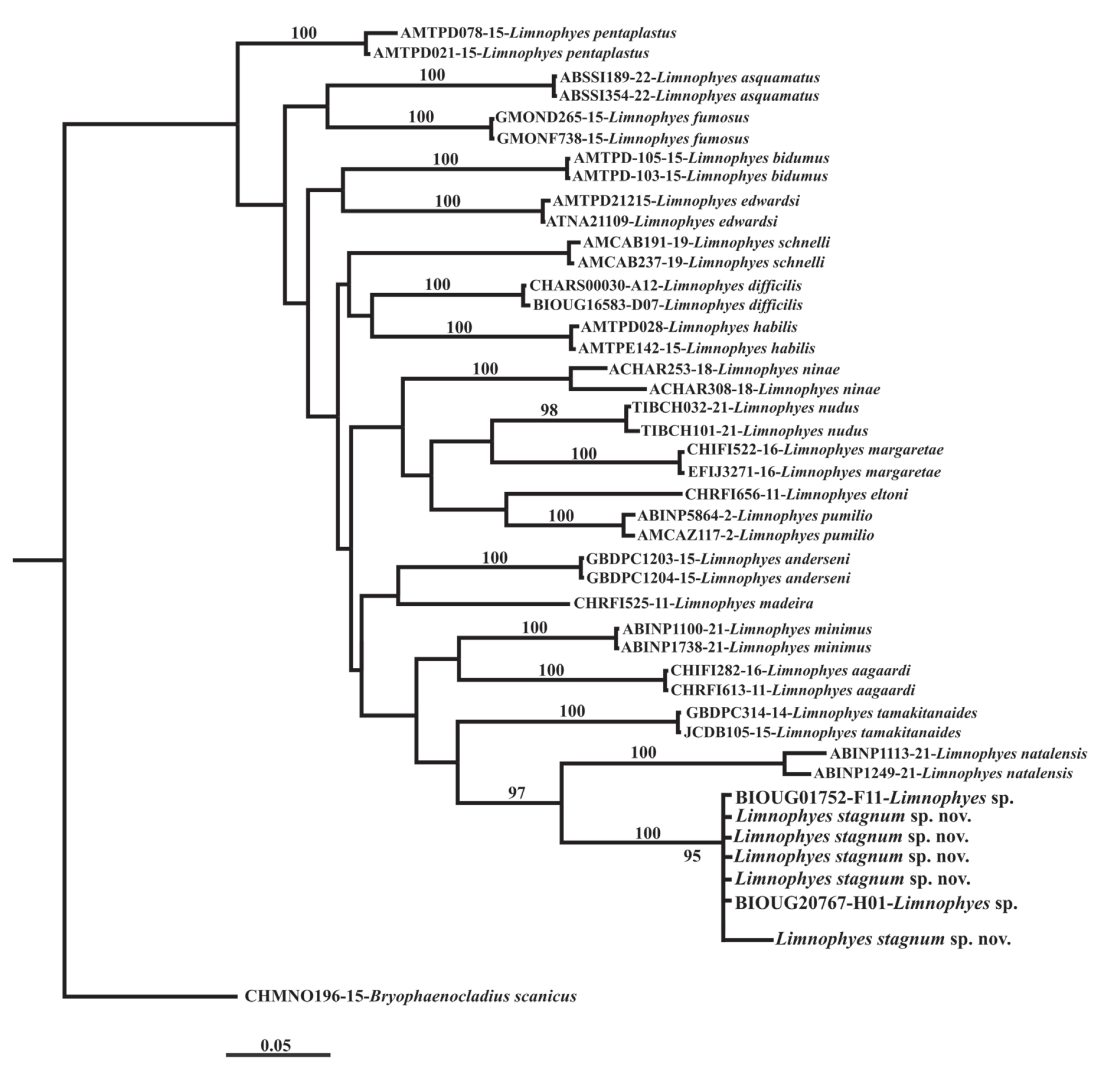
Neighbour-Joining (NJ) and Maximum Likelihood (ML) trees of *Limnophyes* Eaton, 1875 species, and one outgroup *Bryophaenocladiusscanicus* (Brundin, 1947) inferred from the COI nucleotide sequence data (658 bp). Numbers on branches represent the bootstrap value for Neighbor-Joining (NJ) and Maximum Likelihood (10,000 replicates, with values < 95 omitted). Support numbers are equal in both methods.

The analyses of NJ and ML on sequences of *Limnophyes* from Pond A and those obtained from NCBI and BOLD produced the same tree topology (Fig. 3). The six sequences of *L.stagnum* sp. nov., from Pond A, clustered with two sequences identified as *Limnophyes* sp., accession numbers MF727341 and CNTIC4604, from Ontario, Canada (Hebert et al. 2016, de Waard et al. 2019). The average intraspecific K2P distance calculated for the six sequences of *L.stagnum* sp. nov. and the two sequences of *Limnophyes* sp. from Ontario was 0.001 (0.1%). The average interspecific K2P distance of *L.stagnum* sp. nov., with other *Limnophyes* species was 0.17 (17%). The average interspecific K2P distance for all species of *Limnophyes* was 0.15 (15%) (Suppl. material 1: table S3).

**Figure 4.**
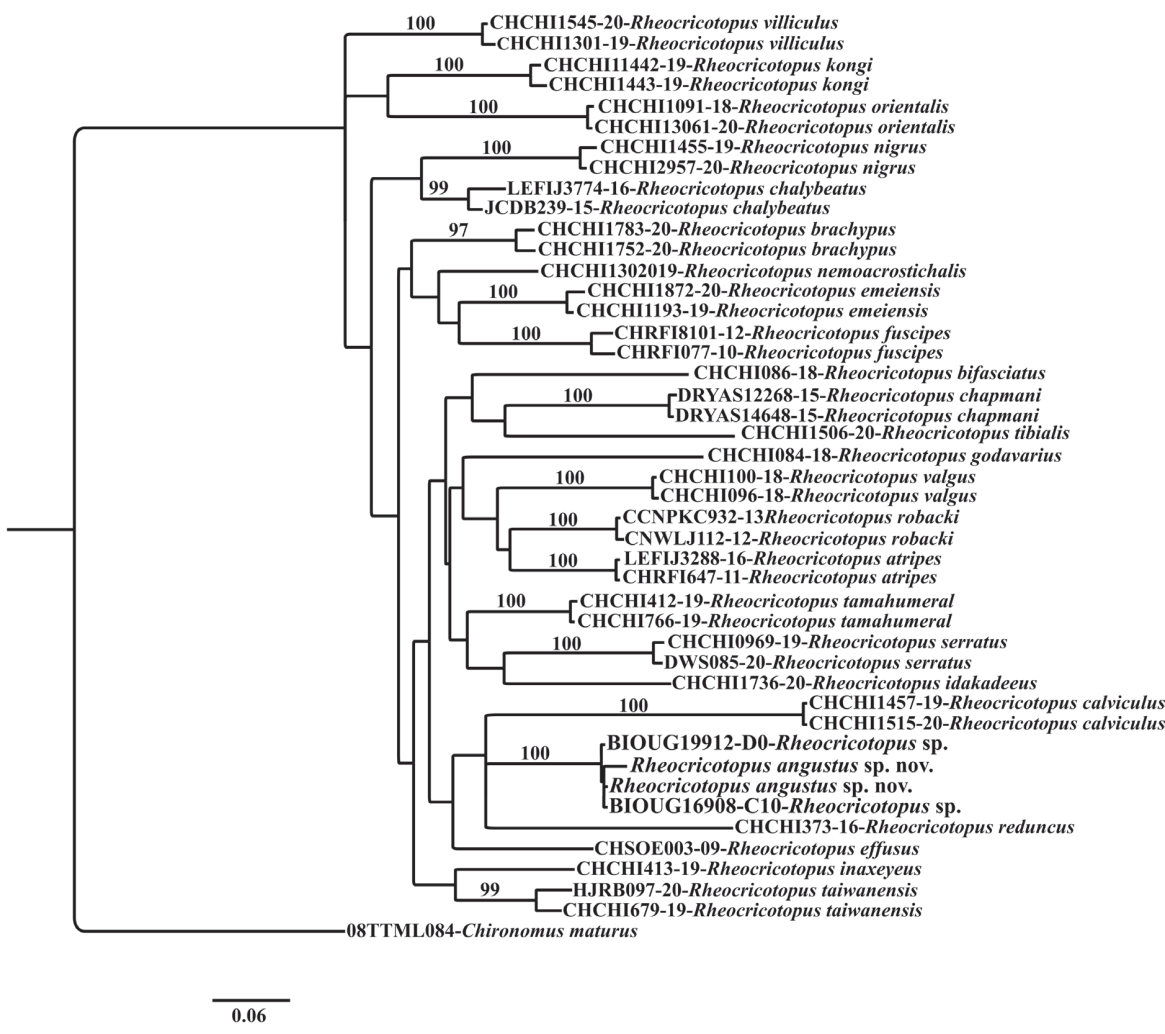
Neighbour-Joining (NJ) and Maximum Likelihood (ML) trees of *Rheocricotopus* Brundin, 1956 species, and one outgroup *Chironomus (s. s.) maturus* Johannsen, 1908 inferred from the COI nucleotide sequence data (658 bp). Numbers on branches represent the bootstrap value for Neighbor-Joining (NJ) and Maximum Likelihood (ML) (10,000 replicates, with values < 95 omitted). Support numbers are equal in both methods.

The analyses of NJ and ML on sequences of *Rheocricotopus* from Pond A and those obtained from NCBI and BOLD produced slightly different tree topologies, with ML more appropriately demonstrating the relationship among species of *Rheocricotopus* (Fig. 4). The two sequences of *R.angustus* sp. nov., from Pond A, clustered with two sequences identified as *Rheocricotopus* sp., accession numbers KR474365.1 and KR470368.1, from Ontario and Yukon Territory, Canada (Hebert et al. 2016). The average intraspecific K2P distance calculated for the two sequences of *R.angustus* sp. nov. with the two sequences of *Rheocricotopus* sp. from Ontario and Yukon was 0.012 (1.2%). The average interspecific K2P distance of *R.angustus* sp. nov. with other *Rheocricotopus* species was 0.16 (16%). The average interspecific K2P distance for all species of *Rheocricotopus* was 0.15 (15%) (Suppl. material 1: tables S4).

**Figure 5.**
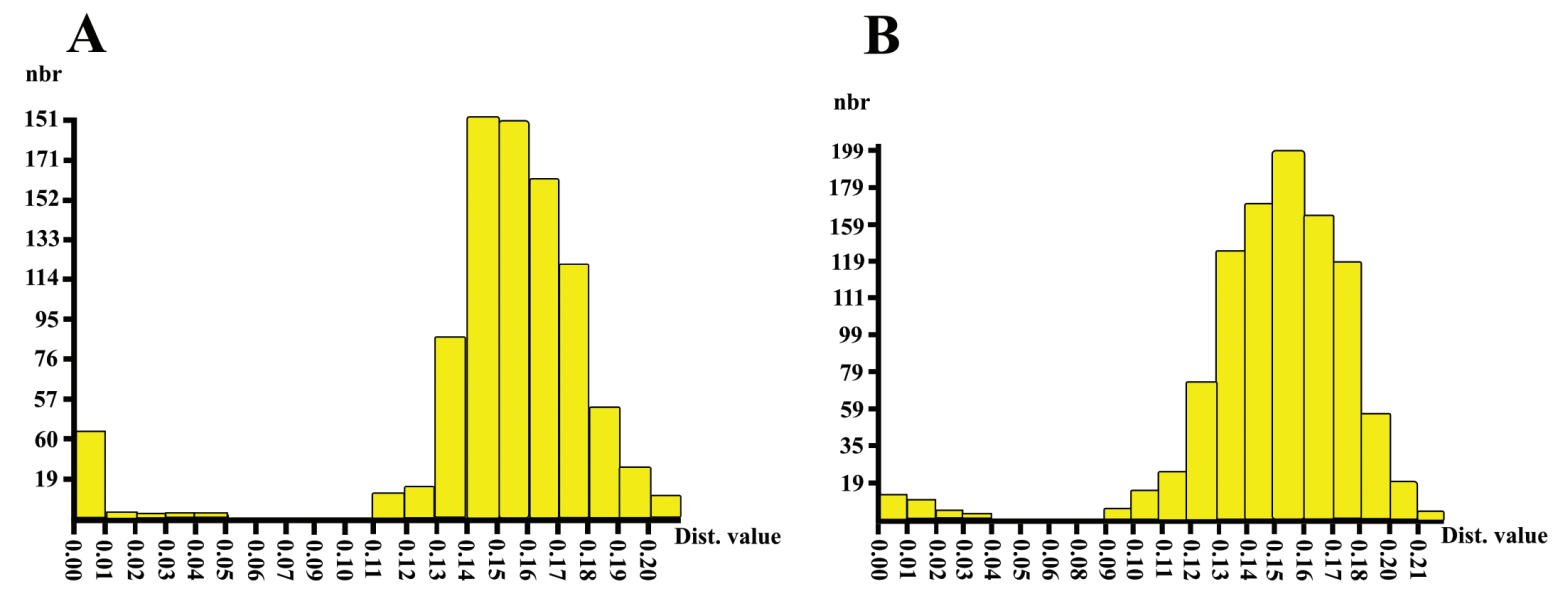
Histogram of genetic distance estimates from ABGD (Automatic Barcode Gap Discovery) for partition analyses **A** 48 cytochrome oxidase subunit1 sequences of the *Limnophyes* Eaton, 1875 species **B** 44 cytochrome oxidase subunit1 sequences of the *Rheocricotopus* Brundin, 1956. nbr = number of runs.

Using ABGD, we saw a gap between the highest intraspecific K2P distance (0.05 or 5%) and the lowest interspecific K2P distance (0.11 or 11%) for *Limnophyes* species. This gap (i.e., for sequences used) suggests that if the distance between two sequences is less than 5%, the sequences belong to the same species, and if it is more than 11%, the sequences belong to two different species. Using ABGD, we also saw a gap between the highest intraspecific K2P distance (0.04 or 4%) and the lowest interspecific K2P distance (0.09 or 9%) for *Rheocricotopus* species. The gaps of 6% and 5% obtained in this study, based on the distance-based methods of ABGD, support species independence (Fig. 5A, B).

### ﻿Faunistic records

**Figure 6.**
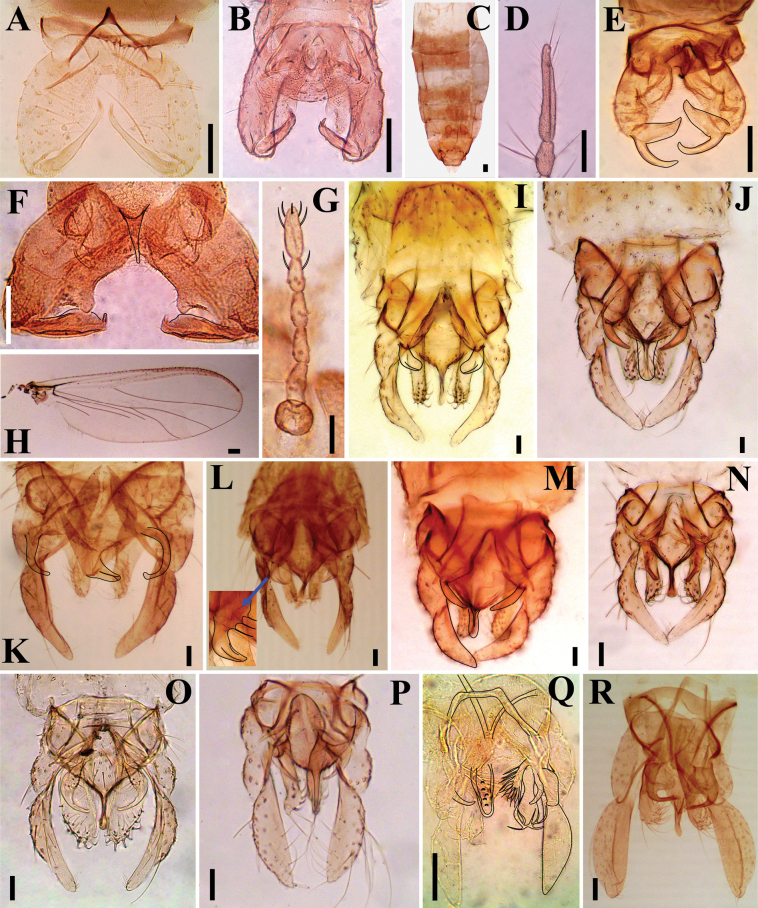
Gallery of Chironomidae species collected from Pond A, 2022–23 **A.***Labrundiniapilosella* (Loew, 1866) **B***Allocladiusnanseni* (Kieffer, 1926) **C, D**Cricotopus (Isocladius) intersectus (Staeger, 1839) **E***Diplosmittiaharrisoni* Sæther, 1981 **F***Smittiaaterima* (Meigen, 1818) **G, H***Smittiaterrestris* Goetghebuer, 1941 **I***Chironomus* (*s. s.*) *acidophilus* Keyl, 1960 **J***Chironomus* (*s. s.*) *bifurcatus* Wülker et al., 2009 **K***Chironomus* sp. ‘*butleri*’ of Martin (2023)**L***Chironomus* (*s. s.*) *maturus* Johannsen, 1908 **M***Chironomus* (*s. s.*) *atrella* (Townes, 1945) **N**Chironomus (Lobochironomus) dorsalis Meigen, 1818 **O***Kiefferulusdux* (Johannsen, 1905) **P***Polypedilum* (*s. s.*) sp. **Q***Tanytarsusguerlus* (Roback, 1957) **R***Tribelosjucundum* (Walker, 1858). , **B, E, F, I–R** male hypopygium **C** female sternites and genitalia **D, G** female antenna **H** female wing. Scale bars: 50 μm.

A total of 20 species of Chironomidae were found in Palmer Park Pond A (Fig. 6; Table 1). Three species *Bryophaenocladiuspalmerparcum* sp. nov., *Limnophyesstagnum* sp. nov., and *Rheocricotopus* (*s. s.*) *angustus* sp. nov., are new to science. *Polypedilum* (*s. s.*) sp. is possibly a new species. *Smittiaterrestris* (Thienemann & Strenzke, 1941) is a new faunistic record for the Nearctic. *Chironomus* (*s. s.*) *acidophilus* Keyl, 1960, *Chironomus* (*s. s.*) *maturus* Johannsen, 1908, Chironomus (Lobochironomus) dorsalis Meigen, 1818, and *Tanytarsusguerlus* (Roback, 1957) are new faunistic records for Michigan.

**Table 1.** List and life stages of species of Chironomidae collected from Palmer Park Pond A, 2022–23. M = Male, F = Female, P = Pupa, L = Larva, N = No, Y = Yes.

Species	Life stage	Barcoded
*Labrundiniapilosella* (Loew, 1866)	1M	N
*Allocladiusnanseni* (Kieffer, 1926)	1M	N
*Bryophaenocladiuspalmerparcum* sp. nov.	3M	N
Cricotopus (Isocladius) intersectus (Staeger, 1839)	1F	N
*Diplosmittiaharrisoni* Sæther, 1981	1M	N
*Rheocricotopus (s. s.) angustus* sp. nov.	3L	Y
*Limnophyesstagnum* sp. nov.	2M, 7F	Y
*Smittiaaterima* (Meigen, 1818)	5M	Y
*Smittiaterrestris* (Thienemann & Strenzke, 1941)	20F	Y
*Chironomus* (*s. s.*) *acidophilus* Keyl, 1960	4M	Y
*Chironomus* (*s. s.*) *bifurcatus* Wülker et al., 2009	1M	N
*Chironomus* sp. ‘*butleri*’ by Martin, 2023	2M	Y
*Chironomus* (*s. s.*) *maturus* Johannsen, 1908	6M, 2F, 1P, 33L	Y
*Chironomus* (*s. s.*) *atrella* (Townes, 1945)	2M	Y
Chironomus (Lobochironomus) dorsalis Meigen, 1818	2M	N
Chironomusnr.sp.parariparius by Martin 2023	1M	N
*Kiefferulusdux* (Johannsen, 1905)	5M	N
*Polypedilum* (*s. s.*) sp.	10M	Y
*Tanytarsusguerlus* (Roback, 1957)	6M	Y
*Tribelosjucundum* (Walker, 1858)	4M	N

### ﻿Taxonomy

**Subfamily Orthocladiinae**

#### ﻿ Bryophaenocladius palmerparcum

Taxon classificationAnimaliaDipteraChironomidae

Namayandeh & Hudson sp. nov.

AEA57F17-2262-5230-AFA0-B36F70AD9613

https://zoobank.org/E9668141-118E-4C08-A504-A65A97E08615

Fig. 7A–D

##### Type material.

***Holotype*** 1 male; USA, Michigan, Detroit, Palmer Park, Pond A; 42.42766°N, 83.11741°W; leg. P.L. Hudson; 30.vi.2022, dep. ARC. ***Paratypes*** 2 males; same as holotype except leg. A. Namayandeh; 28.vi.2023, dep. ARC.

##### Diagnostic characters.

The adult male of *B.palmerparcum* sp. nov. can be distinguished from other *Bryophaenocladius* Thienemann, 1934 males by the combination of the following characters: AR 1.4–1.5; costa not well-extended; anal point short, wide, and triangular, not surpassing the apex of tergite IX, with 11–15 setae; virga inconspicuous, consists of two long spines, looped; sternapodeme straight without large oral projections; superior volsella collar shaped; inferior volsella absent; gonostylus straight with a mid-section wide, and short collar-shaped crista dorsalis; HR 1.5–1.9; HV 2.6–2.9.

##### Description.

**Male** (*n* = 3; unless otherwise stated). Total length 2.7–3.4 mm, Wing 1.4–1.7 mm long and 0.4 mm wide.

***Coloration*.** Head, abdomen, and halter dark brown. Thorax dark brown with much paler yellowish areas in portion of dorsocentrals, in anteprontal, anepisternum, and apical portion of preepisternum regions. Legs golden brown. Wing greyish brown.

**Figure 7.**
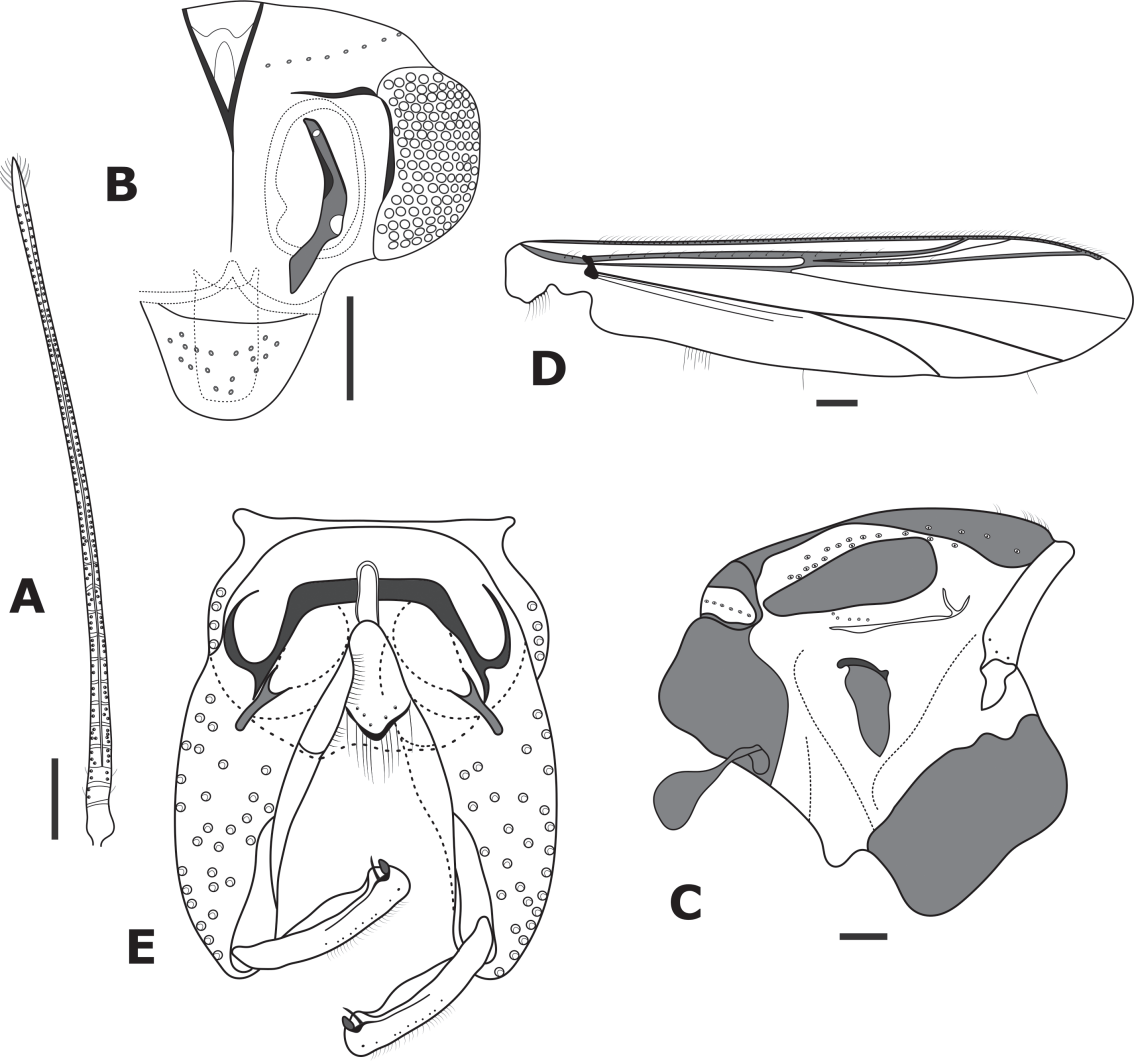
*Bryophaenocladiuspalmerparcum* sp. nov., adult male **A** antenna **B** head **C** thorax **D** wing **E** hypopygium. Scale bars: 100 µm.

***Head*** (Fig. 7A, B). Antenna with 13 flagellomere, last flagellomere with 10 sensilla chaetica, each of 2^nd^ and 3^rd^ flagellomere with pair of sensilla chaetica; shaft starts at base of 4^th^ flagellomere (Fig. 7A); AR 1.4–1.5. Temporal setae 9, uniserial. Tentorium 150–190 µm long, tentorial pit close to apex. Clypeus squared, 101 µm long and 125 µm wide, with 20 setae, setae 73 µm long (*n* = 1). Palpal segments lengths (in μm): 55–77, 66; 33–57, 45; 157–164, 161; 125–131, 128; 93, third palpomere with 3 sensilla clavata, and without any projection.

***Thorax*** (Fig. 7C): Achrostichals 8–10, starting close to antepronotum; dorsocentrals 12–23, 18 in two rows; prealars 4–6, 5; scutellars 12; antepronotals 3 basoventrally.

***Wing*** (Fig. 7D): Brachiolum with 2 setae; R with 7–13, 10 setae; R_1_ with 4 setae; R_4+5_ with 4 setae; other veins bare. Squama with 7–8 setae. Anal lobe produced and squared. Costa not well-extended, 17–20, 18 µm long. Microtrichia visible at 10 × magnification.

***Legs*.** Foreleg spur 58 μm long (*n* = 1), midleg spur 30 μm long (*n* = 1), other spur damaged; hindleg spurs damaged, hind tarsus missing, hindleg comb with 12 spines (*n* = 1). Lengths and proportions of legs as in Table 2.

**Table 2.** Male leg lengths (μm) and proportions of *Bryophaenocladiuspalmerparcum* sp. nov.

	fe	ti	ta_1_	ta_2_	ta_3_	ta_4_	ta_5_	LR	BV	SV
**P_1_**	627	748	729	252	169	116	81	0.97	3.4	1.9
**P_2_**	718	805	391	214	142	104	73	0.50	–	0.50
**P_3_**	828	990	–	–	–	–	–	–	–	–

***Hypopygium*** (Fig. 7E): Anal point short, wide, and triangular, not surpassing the apex of tergite IX, 18 μm long and 34 μm wide at the base (*n* = 1), surface with 11–15, 12 setae. Virga inconspicuous, consists of two long spines, looped, 58 μm long (*n* = 1). Sternapodeme straight with large oral projections, 84–104, 97 μm long. Phallapodeme 42–50, 45 μm long. Superior volsella collar shaped, inferior volsella absent. Gonocoxite 158–216, 187 μm long. Gonostylus straight with a mid-section wide and short collar-shaped crista dorsalis, gonostylus 102–114, 110 μm long, megaseta 9 μm long. HR 1.5–1.9, 1.7; HV 2.6–2.9, 2.8.

**Female and immatures.** unknown.

##### Etymology.

The species is named after the locality where it is found, Palmer Park. The word *parcum* is Latin, meaning park.

##### Distribution.

USA (Michigan).

##### Remarks.

A combination of strong decumbent achrostichals close to antepronotum; bare wing with strong punctation of microtrichia, and weak lateral spines attached to the shaft of hind and mid legs spines places this species in *Bryophaenocladius*. Although, in general, the long, prominent, and hyaline anal point defines many known species of *Bryophaenocladius*, the character of the short anal point of this species is not uncommon, and it has been observed among other known species of this genus. Previously, Donato et al. (2024) have demonstrated that the species of *Bryophaenocladius* show a significant pattern of anal point size and shape differences. For instance, among those species with short anal points, *Bryophaenocladiusscleras* Wang, Liu & Epler, 2012 from the Nearctic has a short semicircular anal point (see Wang et al. 2004), and *Bryophaenocladiuspleuralis* (Malloch, 1915) has a dark short anal point that does not extend beyond tergite IX (Makarchenko and Makarchenko 2009). Additionally, Epler (2012) described brachypterous *Bryophaenocladiuschrissichuckorum* with a wide and short, triangular anal point similar to that of *B.palmerparcum* sp. nov. The first author has also observed this character of short anal point in Neotropical species (AN pers. obs. of specimens from Costa Rica). What is also observable is that besides the variation in shape and size of the anal point, as discussed in Donato et al. (2024), those species with the short anal point can still possess the hyaline section, such as *Bryophaenocladiusinconstans* Brundin, 1947 and some lacking, such as the case of *B.palmerparcum*, *B.chrissichuckorum* and *B.pleuralis*. Therefore, the presence of an anterior hyaline section of the anal point can also separate species with a reduced anal point.

#### ﻿ Limnophyes stagnum

Taxon classificationAnimaliaDipteraChironomidae

Namayandeh, Guerra & Ram sp. nov.

877B68D2-BDD1-5340-A3E3-E1E75489F301

https://zoobank.org/38F6ED6F-9E46-4A00-93EE-0110BF969C6C

Figs 8 , 9

##### Type material.

***Holotype*** 1 male; USA, Michigan, Detroit, Palmer Park, Pond A; 42.42766°N, 83.11741°W; leg. P.L. Hudson; 29.xi.2023, dep. ARC. ***Paratypes*** 2 males, 3 females; same as holotype.

##### Diagnostic characters.

Adults of this species can be separated from other *Limnophyes* by the combination of the following characteristics: Adults with no thoracic lanceolate setae and 2 prescutellars; adult male without humeral setae, female with single humeral setae; male with 3 epimerons, 1 posterior anepisternum II, 1–2 preepisternals anteriorly parallel to antepronotum and close to anapleural suture; female with 2 posterior anepisternals II; 6 epimeron II; 11 preepisternals which 9 anteriorly clustered horizontally, separated from 2 vertical; male antenna with 10 flagellomeres and AR 0.86; female antenna with 4 flagellomere and AR 0.5; male anal point extremely short, almost receded, wide and triangular with apex rounded and gonostylus expanded evenly from base to apex; female with apodeme lobe not distinct; cercus pediform.

##### Description.

**Male** (*n* = 3, unless otherwise indicated). Total length 1.8–1.9 mm. Wing 0.91–1.1 mm long and 0.3 mm wide.

***Coloration*.** Head, thorax, legs, tergites, sternites, and hypopygium blackish brown. Wings and halters grey.

**Figure 8.**
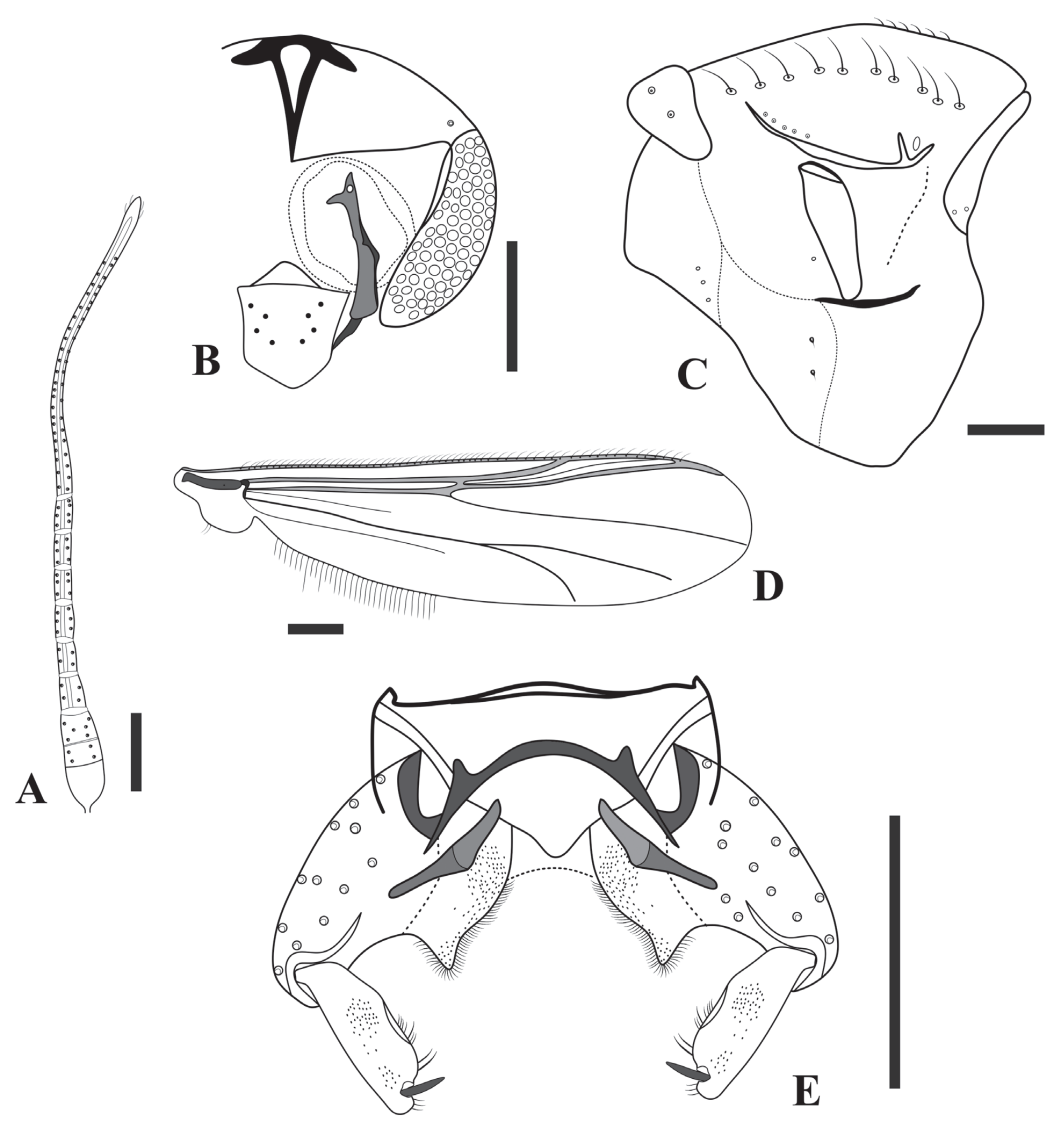
*Limnophyesstagnum* sp. nov., adult male **A** antenna **B** head **C** thorax **D** wing **E** hypopygium. Scale bars: 100 µm.

***Head*** (Fig. 8A, B). Antenna with 10 flagellomeres, last flagellomere with 4 sensilla chaetica, groove starts at the apex of the second segment (Fig. 8A), AR 0.86 (*n* = 1). Eyes bare, without dorsomedial extension. Temporal setae 1 inner vertical (Fig. 8B). Tentorium 113–123, 118 μm long. Clypeus triangular, 82–95, 88 μm long and 106–125, 116 μm wide, bearing 8–10, 9 setae, setae 42–71, 56 μm long. Palpal segment lengths (in μm): 38, 36, 58, 54, 83 (*n* = 1).

***Thorax*** (Fig. 8C). Lanceolate setae absent. Acrostichals 6; dorsocentrals 9–10; prealars 5; scutellars 4 in single row; antepronotals 2; lanceolate humerals absent; 2 prescutellars; epimeron 3; posterior anepisternum II 1; 1–2 preepisternals anteriorly parallel to antepronotum and close to anapleural suture.

***Wing*** (Fig. 8D). Brachiolum with 1 seta. Squama with 2 setae. All veins bare. Costa extension 62 μm long (*n* = 1). Anal lobe well-reduced. Microtrichia visible at 10 ×.

***Legs*.** Fore tibia spur 36–45, 40 μm long, mid tibia spurs 16 and 14 μm long, hind tibia spurs 31 and 24 μm long, hind tibia comb with around 10 spines. Lengths and proportions of legs as in Table 3.

**Table 3.** Male leg lengths (μm) and proportions of *Limnophyesstagnum* sp. nov.

	fe	ti	ta_1_	ta_2_	ta_3_	ta_4_	ta_5_	LR	BV	SV
**P_1_**	469	541	278	163	104	60	69	0.50	3.3	3.6
**P_2_**	496	471	205	116	69	47	63	0.40	4.0	4.7
**P_3_**	447	515	282	150	118	41	68	0.50	3.3	3.4

***Hypopygium*** (Fig. 8E). Anal point extremely short, almost receded, wide, and triangular with apex rounded; anal point 6–15, 10 μm long and 14–34, 24 μm wide. Virga bifid and short, 11–12 μm long. Transverse sternapodeme with well-developed oral projections; sternapodeme 75–79, 77 μm long. Phallapodeme 28–40, 34 μm long. Inferior volsella small triangular lobe covered in numerous simple setae. Gonostylus expanded evenly from base to apex, 67–73, 70 μm long; crista dorsalis very narrow. Gonocoxite 90–116, 103 μm long. HR 1.2–1.7, 1.5, HV 2.5–2.9, 2.7.

**Female** (*n* = 3). Total length 1.7–1.8 mm. Wing 0.96 mm long, 0.38 mm wide.

***Coloration*.** Same as the male.

**Figure 9.**
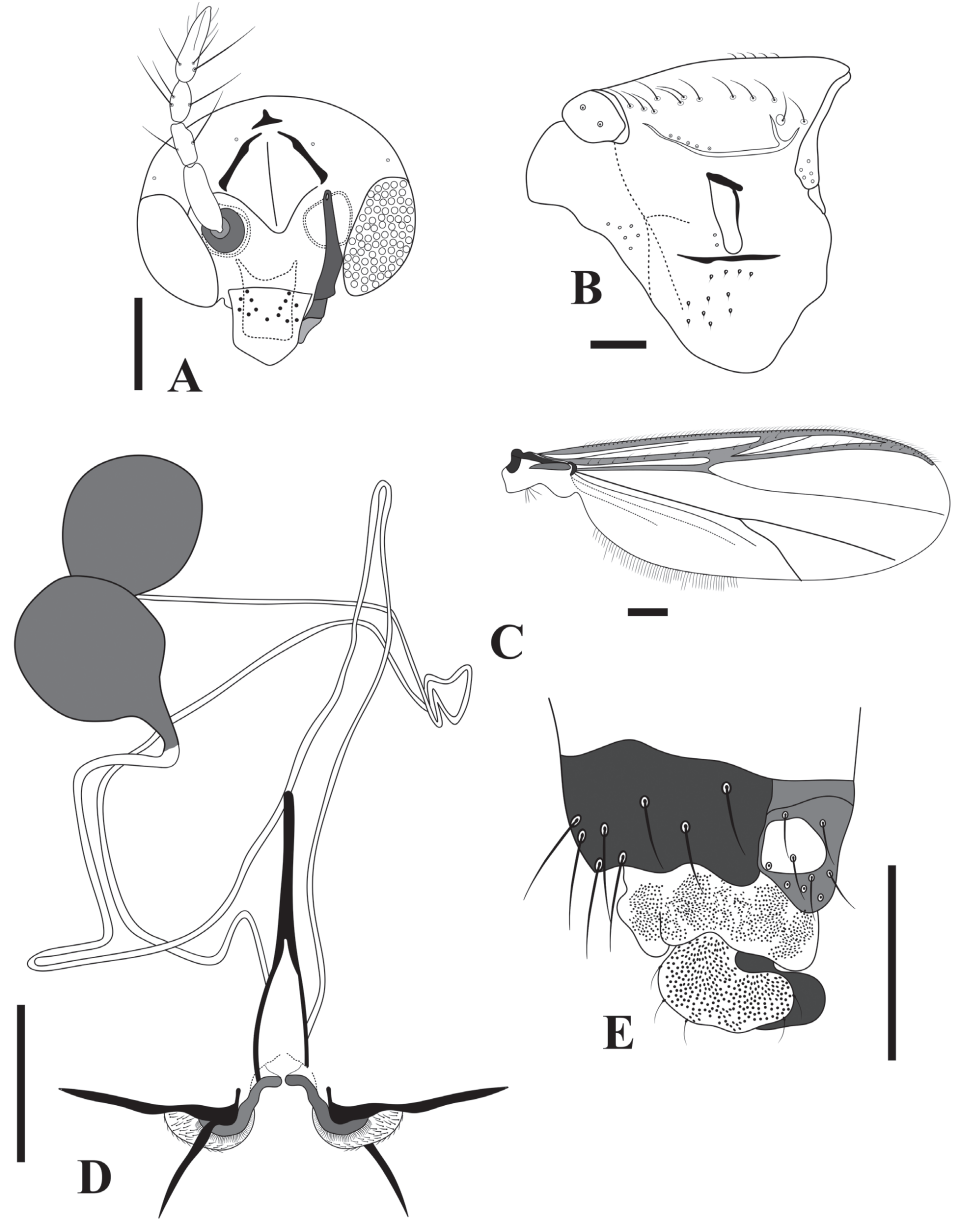
*Limnophyesstagnum* sp. nov., adult female **A** head **B** thorax **C** genitalia ventral **E** genitalia dorsal. Scale bars: 100 µm.

***Head*** (Fig. 9A). Antenna with 4 flagellomeres, last flagellomere with 4 sensilla chaetica, Antennal segments 1–4 (in μm): 75–77, 76; 49, 47, 87, AR 0.5. Eyes bare. Temporal setae 2, including 1 inner vertical and 1 frontal. Tentorium 141–145, 143 μm long. Clypeus triangular, 59 μm long and 46 μm wide, bearing 13 setae, setae 37–59, 47 μm long. Palpal segment lengths (in μm): 46, 32, 52, 54, 75.

***Thorax*** (Fig. 9B). Acrostichals 5; dorsocentrals 8–12, 9 in a single row; prealars 5; scutellars 4 in single row; 1 humerals, non-lanceolate; 2 prescutellars non-lanceolate; 7 antepronotals; 2 posterior anepisternals II; 6 epimeron II; 11 preepisternals, 9 clustered horizontally close to epimeron, 4 horizontally arching close to anapleural suture.

***Wing*** (Fig. 9C). Brachiolum with 1 seta. Squama bare. R with 3–8, 5 setae, R_1_ with 4–5 setae; R_4+5_ with 9–10 setae; other veins bare. Costa extension 50–74, 62 μm. Microtrichia visible at 10 × magnification.

***Legs*.** Hind and mid femur with keel. Fore tibia spur 22–23 μm long, mid tibia spur 21 μm long, second one broken; hind tibia missing. Lengths and proportions of legs as in Table 4.

**Table 4.** Female leg lengths (μm) and proportions of *Limnophyesstagnum* sp. nov.

	fe	ti	ta_1_	ta_2_	ta_3_	ta_4_	ta_5_	LR	BV	SV
**P_1_**	358	440	224	135	86	51	22	0.50	3.0	3.6
**P_2_**	440	427	190	90	61	39	64	0.40	4.2	4.6
**P_3_**	–	–	–	–	–	–	–	–	–	–

***Genitalia*** (Figs 9D, E). Seminal capsules 62–89, 72 μm long, and 47–90, 71 μm wide, semi-circular; spermathecal very long, with well-developed bulb (Fig. 9D). Notum 76–113, 92 µm long, notum and ramus 143–191, 165 µm long. Gonapophysis VIII divided into ventrolateral and thin dorsomesal lobe (Fig. 9D). Apodeme lobe not distinct. Gonocoxite developed, triangular with around 6–9 setae (Fig. 9E). Tergite IX undivided. Cercus small, pediform, 58–72, 65 µm long, and 42–54, 48 µm wide (Fig. 9E).

**Immatures.** Unknown.

##### Etymology.

The species is named after the habitat where it is found. The word *stagnum* is Latin, meaning pond or pool.

##### Distribution.

Canada (British Columbia, Ontario); USA (Michigan).

##### Remarks.

This is a very unusual *Limnophyes* species; lack of lanceolate setae on both sexes, lack of humeral setae in males, and single humeral setae in females are distinguishing characteristics. However, the Neotropical species *Limnophyesbrachyarthra* (Edwards, 1931) described by Sæther (1990) also lacks lanceolate setae and humerals. The adults of *L.stagnum* sp. nov. can be easily separated from those of *L.brachyarthra* based on the number of antennal flagellomere and AR. Additionally, the chaetotaxy of the thorax varies between the two species, and adult males have gonostylus of different shapes and sizes. According to Sæther (1985) the placement of lanceolate humeral setae in relation to the humeral pit varies among *Limnophyes* species. The lanceolate setae can be in or on the margin of humeral pit, concentrated around or above the pit, or scattered over the humerals. Although the lanceolate setae are missing in *L.stagnum* sp. nov., the humeral seta of the female is located on the pit, similar to that in *L.natalensis*. The shape of the hypopygium of the new species also resembles *Limnophyesnatalensis* Kieffer, 1914 as well as *Limnopyesdifficilis* Brundin, 1947. The adult male of the new species can be separated from the adult males of *L.natalensis* and *L.difficilis* based on the number of antennal flagellomeres, antennal ratio, lack of lanceolate and humeral setae, number and formation of thoracic setation, and bare squama. The adult female of the new species can be separated from the adult females of *L.natalensis* and *L.difficilis* based on the number of humeral setae, number and formation of thoracic setation, bare squama, and size of the notum.

#### ﻿ Rheocricotopus (s. s.) angustus

Taxon classificationAnimaliaDipteraChironomidae

 Namayandeh & Hudson sp. nov.

30ED651F-014B-5287-8F4E-5ABCF5069BE4

https://zoobank.org/6D74F69B-4827-43E9-9F1F-122B9D9A8D1F

Figs 10 , 11 , 12

##### Type material.

***Holotype*** 1 male; Canada, Newfoundland, Terra Nova National Park; Blue Hill Road, 48.598°N, -53.9702°W; leg. E. Perry; 21.v.2013, dep. CBG. Paratypes 2 females, CANADA, Yukon Territory, Ivvavik National Park, 69.169°N, -140.167°W; leg. N. Perry; 29.vi.2014, dep. CBG. ***Paratype*** 1 larva; USA, Michigan, Detroit, Palmer Park, Pond A; 42.42766°N, 83.11741°W; leg. P.L. Hudson; 30.vi.2022, dep. ARC.

##### Additional material examined.

*Rheocricotopus* (*s. s.*) *effusus* (Walker, 1856), 1 male, associated pupa and larval exuviae; USA, South Dakota, Yankton, Ed’s Creek, Gavins Point National Fish Hatchery, leg. P.L. Hudson, det. O.A. Sæther, dep. PLH. *Rheocricotopus* (*s. s.*) *effusus* (Walker, 1856), 1 male, associated pupa and larva exuviae; USA, South Dakota, Yankton, Ed’s Creek, Gavins Point National Fish Hatchery, 11.x.1971, leg. P.L. Hudson, dep. PLH. *Rheocricotopus* (*s. s.*) *effusoides* Sæther, 1985, 1 male, associated pupa and larva exuviae; USA, South Dakota, Yankton, Marne Creek, 12.iii.1972, leg. P.L. Hudson, dep. PLH. *Rheocricotopus* (*s. s.*) *unidentatus* Sæther & Schnell, 1988, 1 larva. GERMANY, Federal State Hessen, Freiensteinau, Nature Park Vogelsberg, (north-east f. Frankfurt), forest spring, 02.iv.2017, leg. T. Bendt, dep. TB. *Rheocricotopus* (*s. s.*) *pauciseta* Sæther, 1969, holotype, 1 male, associated pupa and larval exuviae; CANADA, British Columbia, Marion Lake, University of British Columbia Forestry Farm, Haney, small mountain stream, 15.vii.1967, leg., A. L. Hamilton and O. A. Sæther, dep. CNC, No. 9990.

##### Diagnostic characters.

*R.angustus* sp. nov. can be separated from other *Rheocricotopus* by the combination of the following characteristics: Adults with elongate ellipsoid humeral pits, without smaller basal pit, narrowing at the base for the male. Adult male with AR 1.4, anal point very short with 6 lateral setae, superior volsella with caudomedian projection strongly bent, thick, short and triangular. Adult female with AR 0.29, costa extension 115–119 µm long, notum 207–228 µm long. Fourth instar larva with AR 2.2, SI bifid with equal branches, SII long and thin, 37 μm long, mentum’s cardinal beard with 27 setae, seta submenti very long.

##### Description.

**Male** (*n* = 1). Total length 3.3 mm. Wing 1.8 mm long and 0.55 mm wide.

***Coloration*.** Head, thorax, halters, legs, tergites, sternites, and hypopygium blackish brown. Wings pale brown.

**Figure 10.**
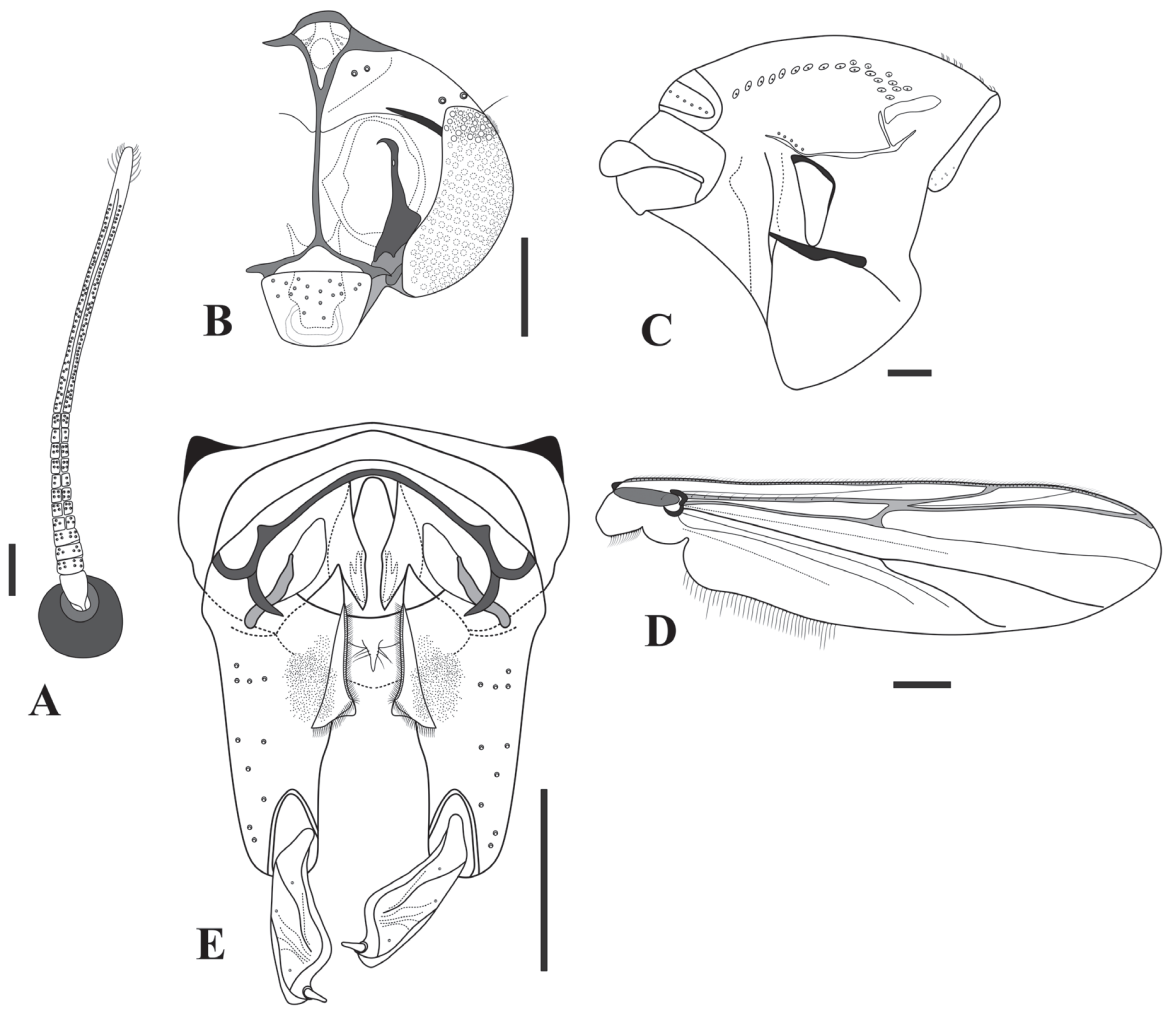
*Rheocricotopus* (*s. s.*) *angustus* sp. nov., adult male **A** antenna **B** head **C** thorax **D** wing **E** hypopygium. Scale bars: 100 µm.

***Head*** (Fig. 10A, B). Antenna with 13 flagellomeres, last flagellomere with 14 sensilla chaetica, groove starts at 4^th^ flagellomere (Fig. 10A), AR 1.4 (*n* = 1). Eyes hairy, without dorsomedial extension. Temporal setae 5, including 2 frontals, 2 postoculars and 1 outer vertical. Tentorium 178 μm long (Fig. 10B). Clypeus rectangular, 89 μm long and 136 μm wide, bearing 18 setae, setae 56–65, 60 μm long. Palpal segment lengths (in μm): 97, 69, 122, 135, 190. Third palpomere with single thin sensilla clavata.

***Thorax*** (Fig. 10C). Acrostichals 12, start close to antepronotum; dorsocentrals 19–20; prealars 5; scutellars 12 in single row; antepronotals 7. Humeral pit elongate ellipsoid narrowing at the base, about half the size of antepronotum, and no smaller basal pit.

***Wing*** (Fig. 10D). Brachiolum with 1 seta. Squama with 13 setae. R with 10 setae; all other veins bare. Costa extension is 44 μm long. Anal lobe developed.

***Legs*.** Fore tibia spur and tarsal segments missing, mid tibia spurs 26 and 22 μm long, hind tibia spurs 55 and 17 μm long, hind tibia comb with 13 spines. Lengths and proportions of legs as in Table 5.

**Table 5.** Male leg lengths (μm) and proportions of *Rheocricotopus* (*s. s.*) *angustus* sp. nov.

	fe	ti	ta_1_	ta_2_	ta_3_	ta_4_	ta_5_	LR	BV	SV
**P_1_**	824	857	–	–	–	–	–	–	–	–
**P_2_**	767	782	375	219	144	95	104	0.50	3.4	4.1
**P_3_**	739	912	516	291	229	121	116	0.60	2.9	3.2

***Hypopygium*** (Fig. 10E). Laterosternite IX 62 μm long, with 12 setae. Anal point very short, 19 μm long and 14 μm wide at the base, triangular with apex pointed and with 6 lateral setae. Transverse sternapodeme with well-developed oral projections; sternapodeme 135 μm long. Phallapodeme 48 μm long. Superior volsella with caudomedian projection tick, short and triangular, parallel but not touching in middle, strongly bent, 35 μm long. Inferior volsella triangular lobe covered in numerous simple setae, apex slightly bent. Gonocoxite 208 μm long. Gonostylus 98 μm long; crista dorsalis very large covering more than half of gonostylus, mega setae 12 μm long. HR 2.1, HV 3.4.

**Female** (*n* = 2). Total length 1.8–2.5, 2.2 mm. Wing 1.5–1.9, 1.7 mm long and 0.56–0.61, 0.58 mm wide.

***Coloration*.** Same as the male.

**Figure 11.**
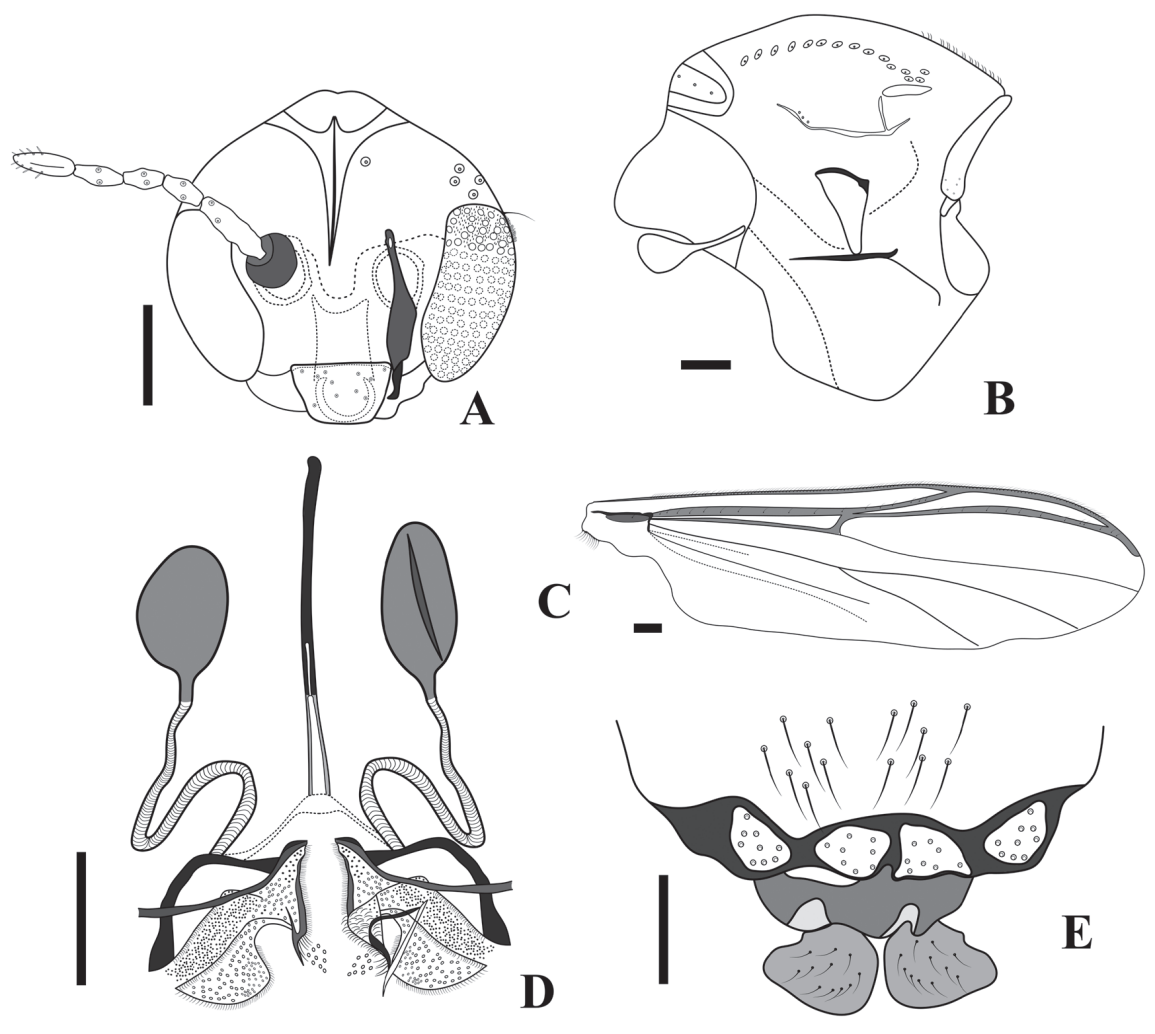
*Rheocricotopus* (*s. s.*) *angustus* sp. nov., adult female **A** head **B** thorax **C** wing **D** genitalia ventral **E** genitalia dorsal. Scale bars: 100 µm.

***Head*** (Fig. 11A). Antenna with 5 flagellomeres, last flagellomere with 6 sensilla chaetica, Antennal segments 1–5 (in μm): 71–101, 86; 42–55, 48; 39–48, 44; 62; 78; AR 0.29. Eyes hairy, reniform. Temporal setae 5 including 1 frontal, 2 outer verticals, and 2 postoculars. Tentorium 144–174, 159 μm long. Clypeus rectangular, 65–82, 74 μm long and 96–110, 104 μm wide, bearing 12 setae, setae 53–63, 59 μm long. Palpal segment lengths (in μm): 27–31, 29; 31–34, 32; 57–71, 64; 61; 72–90, 81.

***Thorax*** (Fig. 11B). Acrostichals 21; dorsocentrals 12–18, 15, in a single row; prealars 3; scutellars 6 in single row; antepronotals 5. The humeral pit is similar to that of the male but not narrow at the base.

***Wing*** (Fig. 11C). Brachiolum with 1 seta. Squama with 10 setae. R with 10 setae; R_1_ with 5–8, 7 setae; R_4+5_ 9–14, 12 setae; other veins without setae. Costa extension 115–119, 117 μm.

***Legs*.** Fore tibia spur missing, mid tibia spurs 17 and 21 μm long, hind tibia spurs 17 and 36 μm long; hind tibia comb with around 13 spines. The lengths and proportions of the legs are shown in Table 6.

**Table 6.** Female leg lengths (μm) and proportions of *Rheocricotopus* (*s. s.*) *angustus* sp. nov.

	fe	ti	ta_1_	ta_2_	ta_3_	ta_4_	ta_5_	LR	BV	SV
**P_1_**	491	513	–	–	–	–	–	–	–	–
**P_2_**	576	522	213	130	98	59	75	0.41	3.6	5.2
**P_3_**	610	666	–	–	–	–	–	–	–	–

***Genitalia*** (Fig. 11D, E). Seminal capsules ovoid, 82–119, 102 μm long, and 56–80, 66 μm wide; spermathecal ducts without loop, with well-developed bulb. Notum 207–228, 218 µm long, notum and ramus 256–285, 270 µm long. Gonapophysis VIII divided into large ventrolateral covering smaller dorsomesal lobe. Apodeme lobe distinct. Gonocoxite developed, with around 8 setae (Fig. 11E). Tergite IX divided in two pale ovoid sections each bearing around 8–9 setae. Cercus large, base semi-circular, apex pediform, 66–89, 78 µm long, and 71–96, 83 µm wide.

**Immatures.** The pupa is unknown. The larva is associated by molecular DNA-barcoding.

4^th^ instar larva (*n* = 1). Total length 7.1 mm. Head 354 μm long and 381 μm wide.

***Coloration of the mounted specimen*.** Head capsule yellowish brown with occipital region darker than rest of the head capsule, body greyish brown.

**Figure 12.**
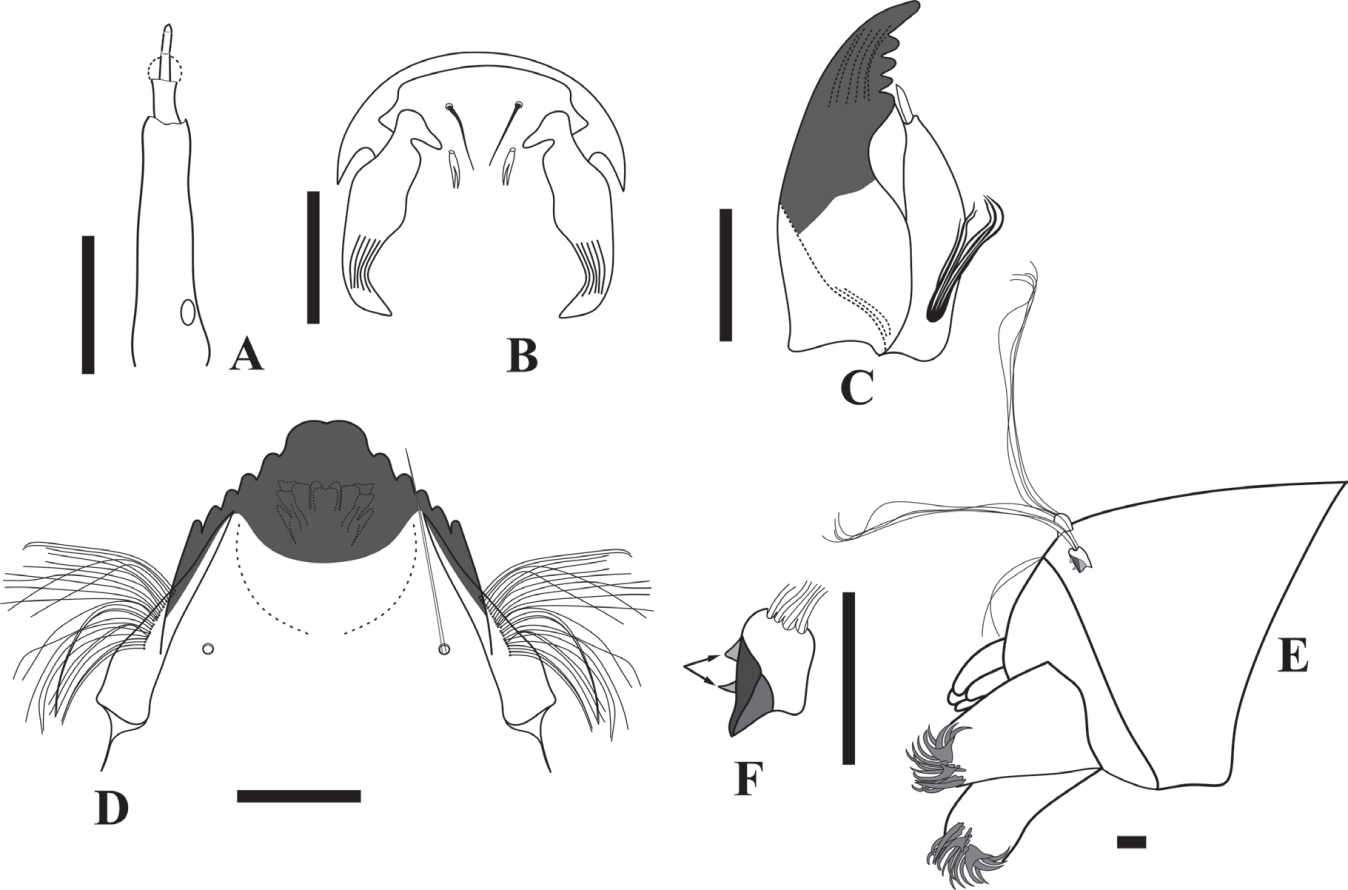
*Rheocricotopus* (*s. s.*) *angustus* sp. nov., fourth instar larva **A** antenna **B** labrum and premandible **C** mandible **D** mentum **E** posterior portion of the larva **F** procercus, arrows indicate the tubercles. Scale bars: 50 µm.

***Head*** (Fig. 12A–D). Antenna 5 segmented; segments length in μm: 86, 14, 10, 7, 7; AR 2.2; basal antennal segment 20 μm wide, distance from the ring organ to base of basal segment 8 μm. Lauterborn organ robust covering the 3^rd^ segment, blade damaged. Labral SI bifid with equal branch, SII–SIII simple, SII long and thin (Fig. 12B). Premandible simple, 78 μm long (Fig. 12B). Mandible much paler in basal half, apical tooth shorter than combined width of three inner teeth; seta subdentalis reaches the base of first inner tooth; setae interna with several long branches (Fig. 12C), mandible 137 μm long. Mentum dark, with large median tooth and 5 pairs of lateral teeth, median tooth slightly notched (possibly worn off), median tooth 35 μm wide, 3.6 × the 1^st^ lateral teeth; seta submenti very long, upright reaching the base of median tooth, base aligned with the 4^th^ inner teeth (Fig. 12D); mentum 92 μm long and 134 μm wide; ventromental plate large, extended beyond the mentum, 116 μm long, and 29 μm wide, cardinal beard with 27 lateral setae. Postmentum 242 μm long.

***Abdomen*** (Fig. 12E, F). Posterior parapods 301 μm long and 208 μm long, bearing around 15 simple claws. Procercus 34 μm long and 18 μm wide, bearing two small basal spurs and 4 apical setae (Fig. 12F), apical setae 447 μm long, supraanal setae 49 μm long. 4 anal tubules present, 78–92 μm long.

##### Etymology.

The new species is named after the city of Detroit. The name of the city comes from the French word *détroit* meaning strait or river, which translates to *angustus* in Latin.

##### Distribution.

Canada (Manitoba, New Brunswick, Newfoundland, Nunavut, Ontario, Yukon Territory); USA (Michigan).

##### Remarks.

Based on the shape of superior volsella, *R.angustus* sp. nov. belongs to the *effusus* group. A detailed examination and comparison of the species in this group is provided by Namayandeh and Beresford (2018). Here, we mentioned that the elongate ellipsoidal shape of the humeral pit of *R.angustus* sp. nov. adults are quite distinguishable from other known species in this group, except *Rheocricotopus* (*s. s.*) *unidentatus* Sæther & Schnell, 1988. However, in *R.angustus* sp. nov., no additional smaller basal pit is present. Another distinguishing characteristic of *R.angustus* sp. nov. male is the very short anal point of the male with few lateral setae. The female of *R.angustus* sp. nov. can be separated from the known females of this group by a lower antennal ratio and longer notum and costa extension.

The key to adult males of the *effusus* group we provided in this study is based on a previous key by Namayandeh and Beresford (2018), which was based on a key by Sæther (1985). The provided key can only partially separate the species in this group. *Rheocricotopus* (*s. s.*) *effusoides* Sæther, 1985 and *Rheocricotopus* (*s. s.*) *effusus* (Walker, 1856) can be separated based on the size and ratio in all life stages (Sæther 1985). *R.unidentatus* is quite distinguishable from other species based on the larval mentum characteristic.

The larva of *R.angustus* sp. nov. can be separated from other known larvae in this group by a higher antennal ratio, a bifid SI with equal branches, and SII 37 μm long. Except for *R.unidentatus*, which has a distinguishing single median mental tooth, there are overlapping characteristics of larvae in this group, which makes their separation difficult. These include but are not limited to the length of the head, postmentum, and basal antennal segment, and number cardinal beard setae (Table 7). However, *R.effusus* and *Rheocricotopuspauciseta* Sæther, 1969 are in the lower range of the basal antennal length in comparison to other known larvae of this group. *R.unidentatus* and *R.effusoides* are in the higher range of cardinal beard setae than other species in this group, and *R.angustus* sp. nov. and *R.pauciseta* are in the lower range. We found that the length and shape of labral SII could also distinguish the larvae in this group (see Table 7). We examined the larval procercus of all species in this group, except for the voucher specimen of *R.effusoides*, in which the abdomen was missing, and consulted Mr. Thomas Bendt on the larva of *R.unidentatus* from Germany, which confirms that all larvae possess a pair of small procercal spurs. However, the shape and size of these spurs are very similar (Table 7).

**Table 7.** Comparison of some relevant larval characteristics of *Rheocricotopuseffusus* group. A_1_ = Antennal segment 1, HL = Head length, L = Length, No. = Number, TL = Total length, W = Width; for other abbreviations, see Sæther (1980). All measurements are in µm unless otherwise indicated.

	*R.angustus* sp. nov.	*R.effusus*	*R.effusoides*	*R.pauciseta*	*R.unidentatus*
**TL (mm)**	7.1	4–5.2	5.2	–	4.0–7.2
**HL**	354	430	424–514	400	450–600
**AR**	2.2	1.5–1.8	1.8–2.1	1.55	1.5–2.0
**A_1_ L**	86	45–64	72–85	62–64	69–87
**Basal A_1_ W**	20	–	19–21	13–18	15–24
**Distance from base to RO**	8	5	12–15	6–10	8–15
**SI**	Bifid, even branches	Bifid, uneven branches	Bifid, uneven branches	Bifid*	Bifid, uneven branches
**SII L**	37	14–22	53	40	19–22
**Mentum median tooth**	Bifid	Bifid	Bifid	Bifid	Single
**No. of cardinal beard setae**	27	25–31	32–33	20	28–40
**Postmentum L**	242	218–226	233–259	234	223–249
**Procercus L**	34	26–28	41–45	40	30–38
**Procercus spurs**	Present	Present	–	Present	Present
**No. procercus apical setae**	4	4–5	–	5	5–6
**Apical setae L**	447	160	549–567	354	450–563

* The labral SI of the examined larvae appears bifid. However, the length of the branches is hard to detect due to the condition of the mount.

### ﻿Key to the adult male of *Rheocricotopuseffusus* group

**Table:** 

1	Superior volsella triangular without distinct caudomedian projection. Inferior volsella distally divided into 2 lobes (Sæther 1969: fig. 47)	**R. (R.) pauciseta Sæther**
–	Superior volsella with distinct caudomedian projection. Inferior volsella simple	**2**
2	Humeral pit small (Namayandeh and Beresford 2018: fig. 2c). Superior volsella with long finger-like caudomedian projection that meet medially (Namayandeh and Beresford 2018: fig. 3b, c)	**3**
–	Humeral pit large. Superior volsella conical with short tapered caudomedian projection	**4**
3	Antennal ratio 0.72–0.79. Anal Point 67 µm long with 12–19 lateral setae (Namayandeh and Beresford 2018: fig. 3b). Caudomedian projection evenly curved (Namayandeh and Beresford 2018: fig. 3c)	**R. (R.) reduncusoides Namayandeh & Beresford**
–	Antennal ratio 0.83–1.17. Anal Point 38 µm long with 6–11 lateral setae (Sæther and Schnell: fig. 3b). Caudomedian projection bent (Namayandeh and Beresford 2018: fig. 4a–c; Sæther and Schnell 1988: fig. 1D)	**R. (R.) reduncus Sæther & Schnell**
4	Anal point very short, 19 µm long, with 6 lateral setae (Fig. 10E, F)	**R. (R.) angustus sp. nov.**
–	Anal point long, ≥ 40 µm long, usually with > 6 lateral setae	**5**
5	Anal point 75–98 µm long, with 15–19 lateral setae (Sæther 1986: fig. 18D). Antennal ratio AR 1.4–1.7	**R. (R.) effusoides Sæther ^1^**
–	Not with the above combination of characters	**6**
6	Dorsocentrals 18–22. Acrostichals 30–36, reaching 26–38 µm in length. Humeral pit elongated ellipsoid, along the axis of antepronotum (Sæther and Schnell 1988: fig. 3B)	**R. (R.) unidentatus Sæther & Schnell ^1^**
–	Dorsocentrals 9–16. Acrostichals 18–26, reaching 15–26 µm in length. Humeral pit large, rounded ellipsoid, perpendicular to antepronotum (Lehman 1969: abb. 13A)	R. (R.) effusus (Walker) ^*^

**Subfamily Chironominae**

#### ﻿ Chironomus nr. sp. parariparius

Taxon classificationAnimaliaDipteraChironomidae

of Martin (2023)

CBD6788B-AF8F-52AC-8D05-EFDEC3D61F79

Figs 13A–E

##### Material examined.

1 male; USA, Michigan, Detroit, Palmer Park, Pond A; 42.42766°N, 83.11741°W; leg. P.L. Hudson; 30.vi.2022, dep. ARC.

##### Diagnostic characters.

The adult male of C.nr.sp.parariparius can be separated from other *Chironomus* species by the combination of the following characteristics: AR 3.1; frontal tubercle present, 12 μm long; wing without any pattern; fore tibia scale 60 μm long; tergite IX with 9 median setae on two adjacent pale patches; superior volsella S-type, with robust apex and base with 5 long setae.

##### Description.

**Male** (*n* = 1). Total length 7.0 mm. Wing 3.6 mm long and 0.90 mm wide.

**Figure 13.**
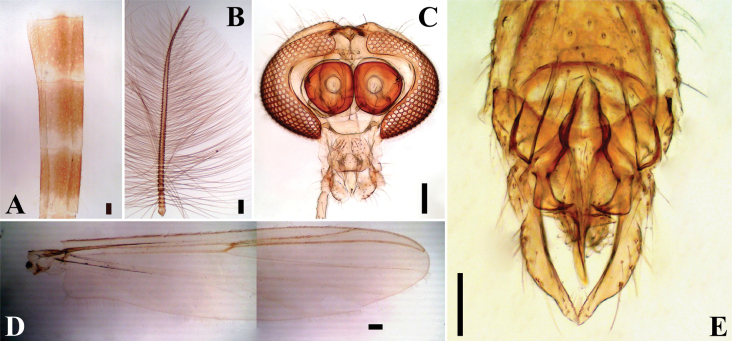
Chironomusnr.sp.parariparius of Martin (2023), adult male **A** abdominal tergites III–V **B** antenna **C** head **D** wing **E** hypopygium. Scale bars: 100 µm.

***Coloration of the mounted specimen*.** Head brown. Thorax brown, with scutellum and humeral region pale yellowish. Abdominal tergites with posterior 2/3^rd^ brown and the anterior 1/3^rd^ pale yellowish (Fig. 13A). Halter and wing pale brown.

***Head*** (Fig. 13B, C). Antenna with 11 flagellomeres, the last flagellomere with 4 sensilla chaetica (Fig. 13B), each of 2^nd^–4^th^ antennal flagellomere with a pair of long sensilla chaetica; groove starts at 4^th^ flagellomere, AR 3.1. Eyes bare, with well-extended dorsomedial extension. Frontal tubercle present, 12 μm long. Temporal setae 25, uniserial. Tentorium 201 μm long. Clypeus nearly squared, 130 μm long and 146 μm wide, bearing 36 setae, setae 108 μm long. Palpal segment lengths (in μm): 216, 116, 272, 234, 337. Third palpomere with 7 sensilla clavata.

***Thorax*.** Acrostichals 2, close to antepronotum; dorsocentrals 14 in single row; prealars 5; scutellars 14 in double rows; antepronotals 1. Mid-scutum hump is present.

***Wing*** (Fig. 13D). Wing without any pattern. Brachiolum with 1 seta, squama with 17 setae, R with 40 setae, R_1_ with 47 setae, R_4+5_ with 65 setae. Anal lobe developed.

***Legs*.** Mid and hind legs missing. Fore tibia scale 60 μm long. The lengths and proportions of the legs as in Table 8.

**Table 8.** Male leg lengths (μm) and proportions of Chironomusnr.sp.parariparius by Martin, 2023.

	fe	ti	ta_1_	ta_2_	ta_3_	ta_4_	ta_5_	LR	BV	SV
**P_1_**	1552	1328	2144	1216	903	722	338	1.6	1.6	1.3
**P_2_**	–	–	–	–	–	–	–	–	–	–
**P_3_**	–	–	–	–	–	–	–	–	–	–

***Hypopygium*** (Fig. 13E). Tergite IX with 12 median setae, seven on adjacent small pale patches and 5 more posteriorly without pale patches, all pointing toward anal point. Anal point broad, parallel-sided with a round apex, 119 μm long. Sternapodeme squared, 129 μm long. Phallapodeme 202 μm long. Superior volsella S-type with robust apex, and base with 5 long setae, 83 μm long. Inferior volsella 178 μm long, base slightly bent, apex reaches the mid-section of the anal point. Gonocoxite 111 μm long. Gonostylus 204 μm long, with 5 long setae at apex; HR 0.5, HV 2.6.

##### Remarks.

The single adult male of the *Chironomus* species we collected from Pond A closely resembles the *Chironomus* species Martin (2023) described as Species 2c. or *Chironomus* species *parariparius*. According to Martin (2023), only the images of the adult male are available based on the reared collection of specimens by J.E. Sublette, now kept in the Zoological Museum of the University of Minnesota, St. Paul. Pupa is only described by the caudolateral spur of segment VIII; however, larva morphology and cytology are described by Martin (2023) in detail. Martin (2023) describes the species’ habitat as snow pools, similar to Pond A. The adult male from pond A is quite distinguishable from other species in the *C.riparius* group based on the characteristics of the hypopygium (Jon Martin, pers. comm. 18 March 2024).

#### ﻿ Polypedilum

Taxon classificationAnimaliaDipteraChironomidae

(s. s.) sp.

DB48793F-1A52-5740-B68A-6C87E885E3B0

##### Remarks.

The adult males of the *Polypedilum* (*s. s.*) specimens that we collected from Pond A resemble *Polypedilum* (*s. s.*) *trigonus*, Townes, 1945. However, these adults did not key out to any known Nearctic adult male in the subgenusPolypedilum based on Maschwitz and Cook (2000). The adult male of this species is distinguished by the following characteristics: head, thorax, and abdomen uniformly dark brown, legs stramineous; AR 1.8; wing unmarked; anal point extending to or slightly higher than inferior volsella; superior volsella without tubercle, sickle-shaped, robust at the base narrowing and bent at apex; inferior volsella tubular and narrow. Based on the molecular data obtained, the species does not match any known *Polypedilum* species in GenBank or BOLD, only to sequences identified only as *Polypedilum* sp., accession numbers HQ982463 and HQ981830. The adult males obtained in this study possibly represent a new species. However, we need to examine more materials, including those of the related species, before we can make the decision on the status of this species. The hypopygium of the adult male is shown in Fig. 6P.

## ﻿Discussion

Vernal pools and, in fact, many isolated or temporary habitats can be easily ignored as insignificant marginal environments. Although ephemeral in their hydrological nature, they are permanent landscape features like other lotic and lentic habitats (Zedler 2003). In an urban environment, these permanent landscape features are likely remnants of a greater ecosystem and, as such, can provide refugia or connecting corridors for a naturally occurring population of species (Dearborn and Kark 2010). Natural areas in city parks surrounded by built-up urban areas can also act as island-like environments in which rapid evolution can occur (Jackson et al. 2022). What we demonstrated in this study is that often overlooked urban natural habitats, such as Palmer Park in a large Detroit metropolitan area, can produce fascinating biological discoveries. These discoveries include new species and faunistic records that would have otherwise been ignored due to a lack of interest in the biodiversity value of urban natural habitats. Further, the discovery of new species and new faunistic records demonstrate the importance of conserving temporary aquatic habitats such as the vernal pools. The public, and in our case, students, are often fascinated with the presence of new species and record discoveries (see DeGasparro et al. 2020). This fascination not only has an educational value for them but also motivates them to engage in the study and conservation of their local natural habitats.

## Supplementary Material

XML Treatment for Bryophaenocladius palmerparcum

XML Treatment for Limnophyes stagnum

XML Treatment for Rheocricotopus (s. s.) angustus

XML Treatment for Chironomus nr. sp. parariparius

XML Treatment for Polypedilum

## Footnotes

See comments in the remarks section.

## ﻿Additional information

### Author ORCIDs

Armin Namayandeh https://orcid.org/0000-0003-2136-0497

Sergio Guerra https://orcid.org/0009-0009-7614-5314

Natasha Islam https://orcid.org/0009-0009-8270-4004

Patrick L. Hudson https://orcid.org/0000-0002-7646-443X

Edris Ghaderi https://orcid.org/0000-0002-9875-7735

Thameena Yusuf https://orcid.org/0009-0000-6296-6693

Adrian A. Vasquez https://orcid.org/0000-0002-2434-3388

Jeffrey L. Ram https://orcid.org/0000-0002-1063-546X

### Data availability

The data associated with the new sequences are available in BOLD database (http://dx.doi.org/10.5883/DS-DTPPA). All other data that support the findings of this study are available in the main text or Supplementary Information.

## Supplementary materials

Supplementary material 1 List of taxa, codes, GenBank, or BOLD accessions Data type pdf https://binary.pensoft.net/file/1103625This dataset is made available under the Open Database License (http://opendatacommons.org/licenses/odbl/1.0/). The Open Database License (ODbL) is a license agreement intended to allow users to freely share, modify, and use this Dataset while maintaining this same freedom for others, provided that the original source and author(s) are credited.Armin Namayandeh, Sergio Guerra, Natasha Islam, Taylor James, Patrick L. Hudson, Edris Ghaderi, Thameena Yusuf, Adrian A. Vasquez, Jeffrey L. Ram
