# Supplementary material for: ﻿New species and a fascinating diversity of Chironomidae (Diptera, Insecta) in and around an overlooked urban vernal pool
Source: Zookeys. 2024 Jul 29;1208:133–63. doi: 10.3897/zookeys.1208.124495 (PMC11301030; doi:10.3897/zookeys.1208.124495)
Supplement: Supplementary material 1 — List of taxa, codes, GenBank, or BOLD accessions [file zookeys-1208-133_article-124495__-s001.pdf]

New species and a fascinating diversity of Chironomidae (Diptera, Insecta) in and around an overlooked urban vernal pool

Armin Namayandeh, Sergio Guerra, Natasha Islam, Taylor James, Patrick L. Hudson, Edris Ghaderi, Thameena Yusuf, Adrian A. Vasquez, Jeffrey L. Ram

TABLE S1. List of taxa, codes, GenBank, or BOLD accessions. Sequences labeled as DTPPA were collected from Palmer Park.

Species	NCBI	BOLD
Bryophaenocladius scanicus	OP927437	GDIP3738-22
Chironomus (s. s.) acidophilus	KR663763	JSJUN1738-11
Chironomus (s. s.) acidophilus	HQ581839	TTMDI362-10
Chironomus (s. s.) atrella	—	DTPPA012-24
Chironomus (s. s.) atrella	HQ582797	USDIQ1587-10
Chironomus (s. s.) atrella	KM995277	CNEIC261-12
Chironomus sp. "*butleri*"	—	DTPPA013-24
Chironomus sp. AAB7030	KT708315	RRINV1261-15
Chironomus (s. s.) maturus	—	DTPPA009-24
Chironomus (s. s.) maturus	—	DTPPA010-24
Chironomus (s. s.) maturus	MF707093	CNRGK640-15
Chironomus (s. s.) maturus	HQ581849	TTMDI373-10
Culicoides sanguisuga	MK760238	BIOUG01687-E07
Limnophyes aagaardi	MZ656362	CHIFI282-16
Limnophyes aagaardi	JF870934	CHRFI613-11
Limnophyes anderseni	—	GBDPC1203-15
Limnophyes anderseni	—	GBDPC1204-15
Limnophyes asquamatus	MN668458	ABSSI189-22
Limnophyes asquamatus	MN668458	ABSSI354-22
Limnophyes bidumus	HQ105113	AMTPD103-1
Limnophyes bidumus	—	AMTPD105-15
Limnophyes difficilis	MN676786	CHARS00030-A12
Limnophyes difficilis	KR434800	BIOUG16583-D07
Limnophyes edwardsi	HM421328	ATNA214-9
Limnophyes edwardsi	HM421325	ATNA211-9
Limnophyes eltoni	JF870974	CHRFI656-11
Limnophyes fumosus	MG303273	GMOND265-15
Limnophyes fumosus	MG305556	GMONF738-15
Limnophyes habilis	—	AMTPD028-15
Limnophyes habilis	—	AMTPE142-15
Limnophyes madeirae	JN275521	CHRFI525-11
Limnophyes margaretae	MZ659181	CHIFI523-16
Limnophyes margaretae	—	EFIJ3271-16
Limnophyes margaretae	MZ656593	CHIFI522-16

New species and a fascinating diversity of Chironomidae (Diptera, Insecta) in and around an overlooked urban vernal pool

Armin Namayandeh, Sergio Guerra, Natasha Islam, Taylor James, Patrick L. Hudson, Edris Ghaderi, Thameena Yusuf, Adrian A. Vasquez, Jeffrey L. Ram

Species	NCBI	BOLD
Limnophyes minimus	—	ABINP1100-21
Limnophyes minimus	—	ABINP1738-21
Limnophyes natalensis	KR656550	ABINP1113-21
Limnophyes natalensis	MN677861	ABINP1249-21
Limnophyes nudus	—	TIBCH028-21
Limnophyes nudus	—	TIBCH032-21
Limnophyes pentaplastus	—	AMTPD021-15
Limnophyes pentaplastus	—	AMTPD078-15
Limnophyes pumilio	—	ABINP5864-2
Limnophyes pumilio	—	AMCAZ117-2
Limnophyes schnelli	—	AMCAB191-19
Limnophyes schnelli	—	AMCAB237-19
Limnophyes sp.	KR695614	NCCA1869-11
Limnophyes sp.	MF727341	CNTIC4604-15
Limnophyes stagnum **sp. nov.**	—	DTPPA003-24
Limnophyes stagnum **sp. nov.**	—	DTPPA004-24
Limnophyes stagnum **sp. nov.**	—	DTPPA005-24
Limnophyes stagnum **sp. nov.**	—	DTPPA006-24
Limnophyes stagnum **sp. nov.**	—	DTPPA007-24
Limnophyes stagnum **sp. nov.**	—	DTPPA008-24
Limnophyes tamakitanaides	—	GBDPC314-14
Limnophyes tamakitanaides	—	JCDB105-15
Polypedilum sp.	—	DTPPA014-24
Polypedilum sp.	—	DTPPA015-24
Polypedilum sp.	HQ982463	TTMDJ693-10
Polypedilum sp.	HQ981830	TTMDI691-10
Rheocricotopus angustus **sp. nov.**	—	DTPPA001-24
Rheocricotopus angustus **sp. nov.**	—	DTPPA002-24
Rheocricotopus atripes	MZ659529	LEFIJ3288-16
Rheocricotopus atripes	JF870965	CHRFI647-11
Rheocricotopus bifasciatus	—	CHCHI086-18
Rheocricotopus brachypus	—	CHCHI1752-20
Rheocricotopus calviculus	MT456605	CHCHI1515-20
Rheocricotopus calviculus	MT456597	CHCHI1457-19
Rheocricotopus chalybeatus	—	JCDB239-15
Rheocricotopus chalybeatus	MZ658471	LEFIJ3774-16

New species and a fascinating diversity of Chironomidae (Diptera, Insecta) in and around an overlooked urban vernal pool

Armin Namayandeh, Sergio Guerra, Natasha Islam, Taylor James, Patrick L. Hudson, Edris Ghaderi, Thameena Yusuf, Adrian A. Vasquez, Jeffrey L. Ram

Species	NCBI	BOLD
Rheocricotopus chapmani	—	DRYAS12268-15
Rheocricotopus chapmani	—	DRYAS14648-15
Rheocricotopus effusus	HM406100	CHSOE003-9
Rheocricotopus emeiensis	—	CHCHI1193-19
Rheocricotopus emeiensis	—	CHCHI1872-20
Rheocricotopus fuscipes	—	CHRFI077-10
Rheocricotopus fuscipes	—	CHRFI810-12
Rheocricotopus godavarius	—	CHCHI084-18
Rheocricotopus idakadeeus	—	CHCHI1736-20
Rheocricotopus inaxeyeus	—	CHCHI413-19
Rheocricotopus kongi	MT456602	CHCHI1442-19
Rheocricotopus kongi	MT456600	CHCHI1443-19
Rheocricotopus nemoacrostichalis	—	CHCHI1302-19
Rheocricotopus nigrus	—	CHCHI1455-19
Rheocricotopus nigrus	—	CHCHI2957-20
Rheocricotopus orientalis	MT456598	CHCHI091-18
Rheocricotopus orientalis	—	CHCHI3061-20
Rheocricotopus reduncus	MZ660090	CHIFI373-16
Rheocricotopus robacki	KR174271	CNPKC932-13
Rheocricotopus robacki	KM935438	CNWLJ112-12
Rheocricotopus serratus	—	DWS085-20
Rheocricotopus serratus	—	CHCHI969-19
Rheocricotopus sp.	KR474365	CNIVD455-14
Rheocricotopus sp.	KR470368	CNIVD844-14
Rheocricotopus taiwanensis	—	HJRB097-20
Rheocricotopus taiwanensis	—	CHCHI679-19
Rheocricotopus tamahumeral	—	CHCHI766-19
Rheocricotopus tamahumeral	—	CHCHI412-19
Rheocricotopus tibialis	—	CHCHI1506-20
Rheocricotopus valgus	—	CHCHI096-18
Rheocricotopus valgus	—	CHCHI100-18
Rheocricotopus villiculus	MT456593	CHCHI1545-20
Rheocricotopus villiculus	MT456604	CHCHI301-19
Rheocricotopush brachypus	—	CHCHI1783-20
Smittia aterima	—	DTPPA017-24
Smittia aterima	KR739737	PHOCT699-11

New species and a fascinating diversity of Chironomidae (Diptera, Insecta) in and around an overlooked urban vernal pool

Armin Namayandeh, Sergio Guerra, Natasha Islam, Taylor James, Patrick L. Hudson, Edris Ghaderi, Thameena Yusuf, Adrian A. Vasquez, Jeffrey L. Ram

Species	NCBI	BOLD
Smittia sp.	MG301977	PHOCT181-11
Smittia sp.	HQ582868	USDIQ1662-10
Smittia terrestris	OP927437	GDIP3738-22
Smittia terrestris	—	DTPPA018-24
Tanytarsus guerlus	KR657911	JSJUL240-11
Tanytarsus guerlus	KR638783	GMDAP080-12
Tanytarsus guerlus	—	DTPPA016-24

New species and a fascinating diversity of Chironomidae (Diptera, Insecta) in and around an overlooked urban vernal pool

Armin Namayandeh, Sergio Guerra, Natasha Islam, Taylor James, Patrick L. Hudson, Edris Ghaderi, Thameena Yusuf, Adrian A. Vasquez, Jeffrey L. Ram

TABLE S2. Kimura 2-Parameter (K2P) average interspecific distances obtained in MEGA X for the species of Chironomidae of Pond A, Plamer Park, Detroit, Michigan, USA.

	C. maturus	***C. atrella***	***C.* sp. "*butleri*"**	***C. acidophilus***	***L. stagnum* sp. nov.**	***P.* sp.**	***R. angustus* sp. nov.**	***T. guerlus***	***S. aterima***	***S. terrestris***
C. maturus										
C. atrella	0.13									
***C.* sp. "*butleri*"**	0.10	0.12								
C. acidophilus	0.15	0.15	0.14							
***L. stagnum* sp. nov.**	0.24	0.25	0.23	0.23						
***P.* sp.**	0.20	0.17	0.16	0.19	0.19					
***R. angustus* sp. nov.**	0.23	0.22	0.19	0.24	0.20	0.21				
T. guerlus	0.18	0.19	0.16	0.18	0.19	0.16	0.16			
S. aterima	1.01	1.04	0.99	1.05	1.03	0.95	1.02	1.00		
S. terrestris	1.12	1.07	1.05	1.11	1.04	0.96	1.00	1.00	0.17	

New species and a fascinating diversity of Chironomidae (Diptera, Insecta) in and around an overlooked urban vernal pool

Armin Namayandeh, Sergio Guerra, Natasha Islam, Taylor James, Patrick L. Hudson, Edris Ghaderi, Thameena Yusuf, Adrian A. Vasquez, Jeffrey L. Ram

TABLE S3. Kimura 2-Parameter (K2P) average interspecific distances obtained in MEGA X for the species of *Limnophyes* Eaton.

	L. difficilis	***L. habilis***	***L. schnelli***	***L. asquamatus***	***L. fumosus***	***L. pentaplastus***	***L. bidumus***	***L. edwardsi***	***L. anderseni***	***L. madeirae***	***L. nudus***	***L. margaretae***	***L. pumilio***	***L. eltoni***	***L. aagaardi***	***L. minimus***	***L. tamakitanaides***	***L. natalensis***	***L. stagnum* sp. nov.**
L. difficilis																			
L. habilis	0.12																		
L. schnelli	0.13	0.15																	
L. asquamatus	0.15	0.13	0.15																
L. fumosus	0.14	0.13	0.16	0.14															
L. pentaplastus	0.13	0.12	0.14	0.13	0.13														
L. bidumus	0.16	0.16	0.17	0.18	0.14	0.13													
L. edwardsi	0.14	0.14	0.15	0.16	0.14	0.15	0.14												
L. anderseni	0.15	0.17	0.14	0.15	0.16	0.15	0.15	0.14											
L. madeirae	0.14	0.15	0.15	0.15	0.15	0.13	0.15	0.15	0.14										
L. nudus	0.14	0.14	0.14	0.17	0.16	0.13	0.14	0.14	0.13	0.15									
L. margaretae	0.14	0.14	0.17	0.18	0.16	0.12	0.17	0.15	0.15	0.15	0.12								
L. pumilio	0.14	0.16	0.14	0.16	0.15	0.14	0.15	0.16	0.14	0.15	0.12	0.15							
L. eltoni	0.16	0.16	0.14	0.14	0.15	0.13	0.15	0.17	0.14	0.15	0.13	0.14	0.11						
L. aagaardi	0.16	0.14	0.13	0.15	0.13	0.14	0.16	0.16	0.15	0.16	0.16	0.18	0.17	0.16					
L. minimus	0.15	0.14	0.15	0.17	0.16	0.16	0.17	0.15	0.16	0.14	0.13	0.17	0.15	0.14	0.13				
L. tamakitanaides	0.15	0.14	0.16	0.17	0.17	0.13	0.14	0.17	0.17	0.17	0.15	0.17	0.14	0.15	0.15	0.14			
L. natalensis	0.17	0.17	0.19	0.17	0.18	0.16	0.17	0.16	0.16	0.18	0.17	0.19	0.18	0.17	0.17	0.16	0.16		
***L. stagnum* sp. nov.**	0.15	0.16	0.18	0.17	0.17	0.17	0.19	0.16	0.16	0.17	0.17	0.18	0.17	0.19	0.15	0.15	0.15	0.15	

New species and a fascinating diversity of Chironomidae (Diptera, Insecta) in and around an overlooked urban vernal pool

Armin Namayandeh, Sergio Guerra, Natasha Islam, Taylor James, Patrick L. Hudson, Edris Ghaderi, Thameena Yusuf, Adrian A. Vasquez, Jeffrey L. Ram

TABLE S4. Kimura 2-Parameter (K2P) average interspecific distances obtained in MEGA X for the species of *Rheocricotopus* Brundin.

	R. reduncus	***R. calviculus***	***R. orientalis***	***R. atripes***	***R. robacki***	***R. valgus***	***R. tamahumeral***	***R. chapmani***	***R. bifasciatus***	***R. serratus***	***R. idakadeeus***	***R. effusus***	***R angustus* sp. nov.**	***R. godavarius***	***R. kongi***	***R. tibialis***	***R. brachypus***	***R. emeiensis***	***R. nemoacrostichalis***	***R. fuscipes***	***R. chalybeatus***	***R. nigrus***	***R. taiwanensis***	***R. inaxeyeus***	***R. villiculus***
R. reduncus																									
R. calviculus	0.17																								
R. orientalis	0.18	0.18																							
R. atripes	0.15	0.18	0.17																						
R. robacki	0.16	0.17	0.17	0.10																					
R. valgus	0.18	0.18	0.18	0.12	0.13																				
R. tamahumeral	0.16	0.19	0.16	0.12	0.10	0.13																			
R. chapmani	0.17	0.18	0.18	0.13	0.15	0.17	0.14																		
R. bifasciatus	0.20	0.19	0.19	0.17	0.16	0.18	0.16	0.15																	
R. serratus	0.16	0.19	0.18	0.14	0.15	0.17	0.12	0.14	0.18																
R. idakadeeus	0.15	0.17	0.19	0.14	0.15	0.16	0.13	0.14	0.17	0.13															
R. effusus	0.15	0.19	0.17	0.15	0.14	0.15	0.12	0.13	0.16	0.16	0.17														
***R. angustus* sp. nov.**	0.15	0.17	0.20	0.13	0.15	0.18	0.15	0.15	0.16	0.15	0.16	0.13													
R. godavarius	0.18	0.18	0.19	0.14	0.14	0.16	0.15	0.17	0.18	0.17	0.16	0.16	0.16												
R. kongi	0.18	0.20	0.16	0.14	0.15	0.18	0.14	0.17	0.18	0.16	0.18	0.14	0.17	0.16											
R. tibialis	0.19	0.20	0.19	0.16	0.17	0.20	0.15	0.15	0.16	0.18	0.19	0.17	0.19	0.18	0.16										
R. brachypus	0.16	0.18	0.16	0.14	0.14	0.15	0.11	0.16	0.16	0.16	0.15	0.14	0.16	0.17	0.15	0.15									
R. emeiensis	0.16	0.17	0.16	0.14	0.13	0.16	0.12	0.17	0.17	0.15	0.16	0.12	0.14	0.16	0.15	0.15	0.12								
R. nemoacrostichalis	0.15	0.19	0.17	0.13	0.13	0.16	0.11	0.14	0.16	0.15	0.16	0.14	0.14	0.18	0.16	0.14	0.11	0.11							
R. fuscipes	0.16	0.17	0.18	0.15	0.15	0.18	0.14	0.15	0.18	0.14	0.16	0.14	0.14	0.19	0.17	0.17	0.13	0.13	0.12						
R. chalybeatus	0.17	0.18	0.16	0.13	0.12	0.17	0.12	0.16	0.18	0.16	0.18	0.13	0.14	0.16	0.13	0.16	0.12	0.11	0.12	0.15					
R. nigrus	0.18	0.19	0.17	0.14	0.13	0.18	0.13	0.17	0.19	0.17	0.18	0.16	0.17	0.18	0.15	0.19	0.13	0.14	0.14	0.14	0.11				

New species and a fascinating diversity of Chironomidae (Diptera, Insecta) in and around an overlooked urban vernal pool

Armin Namayandeh, Sergio Guerra, Natasha Islam, Taylor James, Patrick L. Hudson, Edris Ghaderi, Thameena Yusuf, Adrian A. Vasquez, Jeffrey L. Ram

	R. reduncus	***R. calviculus***	***R. orientalis***	***R. atripes***	***R. robacki***	***R. valgus***	***R. tamahumeral***	***R. chapmani***	***R. bifasciatus***	***R. serratus***	***R. idakadeeus***	***R. effusus***	***R angustus* sp. nov.**	***R. godavarius***	***R. kongi***	***R. tibialis***	***R. brachypus***	***R. emeiensis***	***R. nemoacrostichalis***	***R. fuscipes***	***R. chalybeatus***	***R. nigrus***	***R. taiwanensis***	***R. inaxeyeus***	***R. villiculus***
R. taiwanensis	0.18	0.17	0.18	0.15	0.14	0.18	0.13	0.15	0.15	0.15	0.17	0.14	0.15	0.18	0.15	0.17	0.13	0.13	0.14	0.15	0.13	0.16			
R. inaxeyeus	0.16	0.18	0.18	0.14	0.14	0.16	0.14	0.16	0.16	0.15	0.19	0.12	0.14	0.18	0.15	0.15	0.15	0.12	0.12	0.15	0.11	0.15	0.10		
R. villiculus	0.15	0.19	0.17	0.14	0.13	0.17	0.14	0.15	0.18	0.16	0.18	0.17	0.16	0.16	0.15	0.15	0.13	0.14	0.13	0.15	0.12	0.15	0.13	0.14	

New species and a fascinating diversity of Chironomidae (Diptera, Insecta) in and around an overlooked urban vernal pool

Armin Namayandeh, Sergio Guerra, Natasha Islam, Taylor James, Patrick L. Hudson, Edris Ghaderi, Thameena Yusuf, Adrian A. Vasquez, Jeffrey L. Ram

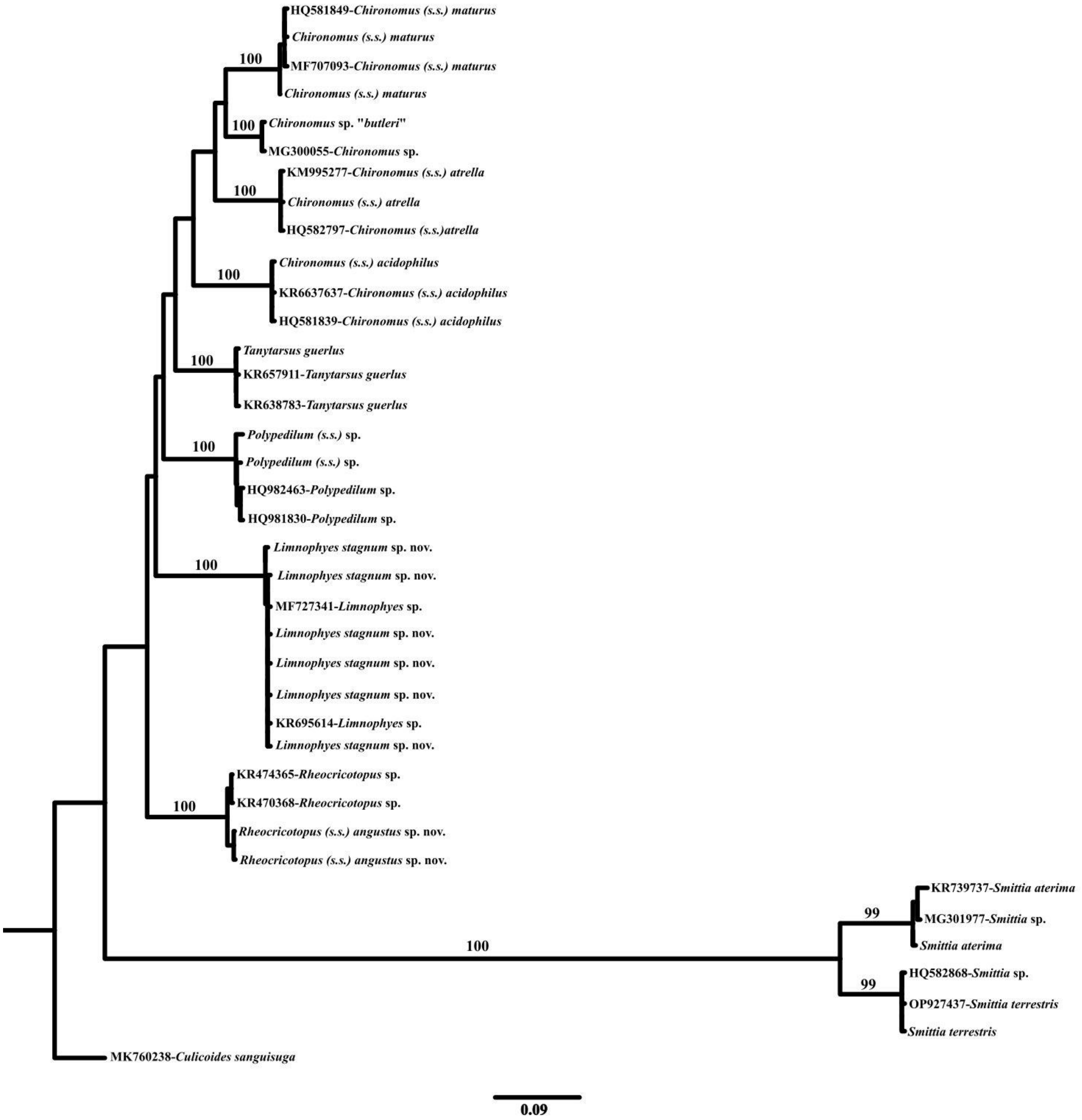

Fig. S1. Neighbor-Joining (NJ) tree of the Palmer Park Pond A Chironomidae, and one outgroup *Culicoides sanguisuga* Gornostaeva, 1977 inferred from the COI nucleotide sequence data (658 bp). Numbers on branches represent the bootstrap value for Neighbor-Joining (NJ) (10000 replicates, with values < 95 omitted).
